# Collaborative Equilibrium Coupling of Catalytic DNA Nanostructures Enables Programmable Detection of SARS‐CoV‐2

**DOI:** 10.1002/advs.202101155

**Published:** 2021-07-18

**Authors:** Yuan Chen, Noah R. Sundah, Nicholas R. Y. Ho, Auginia Natalia, Yu Liu, Qing Hao Miow, Yu Wang, Darius L. L. Beh, Ka Lip Chew, Douglas Chan, Paul A. Tambyah, Catherine W. M. Ong, Huilin Shao

**Affiliations:** ^1^ Institute for Health Innovation & Technology National University of Singapore Singapore 117599 Singapore; ^2^ Department of Biomedical Engineering Faculty of Engineering National University of Singapore Singapore 117583 Singapore; ^3^ Institute of Molecular and Cell Biology Agency for Science Technology and Research Singapore 138673 Singapore; ^4^ Department of Medicine Yong Loo Lin School of Medicine National University of Singapore Singapore 117599 Singapore; ^5^ Division of Infectious Diseases Department of Medicine National University Hospital Singapore 119074 Singapore; ^6^ Department of Laboratory Medicine National University Hospital Singapore 119074 Singapore; ^7^ Department of Laboratory Medicine Ng Teng Fong General Hospital Singapore 609606 Singapore; ^8^ Department of Surgery Yong Loo Lin School of Medicine National University of Singapore Singapore 117599 Singapore

**Keywords:** catalytic DNA nanostructures, collaborative equilibrium coupling, direct and programmable detection, molecular nanotechnology, nucleic acid testing

## Abstract

Accessible and adaptable nucleic acid diagnostics remains a critical challenge in managing the evolving COVID‐19 pandemic. Here, an integrated molecular nanotechnology that enables direct and programmable detection of SARS‐CoV‐2 RNA targets in native patient specimens is reported. Termed synergistic coupling of responsive equilibrium in enzymatic network (SCREEN), the technology leverages tunable, catalytic molecular nanostructures to establish an interconnected, collaborative architecture. SCREEN mimics the extraordinary organization and functionality of cellular signaling cascades. Through programmable enzyme–DNA nanostructures, SCREEN activates upon interaction with different RNA targets to initiate multi‐enzyme catalysis; through system‐wide favorable equilibrium shifting, SCREEN directly transduces a single target binding into an amplified electrical signal. To establish collaborative equilibrium coupling in the architecture, a computational model that simulates all reactions to predict overall performance and optimize assay configuration is developed. The developed platform achieves direct and sensitive RNA detection (approaching single‐copy detection), fast response (assay reaction is completed within 30 min at room temperature), and robust programmability (across different genetic loci of SARS‐CoV‐2). When clinically evaluated, the technology demonstrates robust and direct detection in clinical swab lysates to accurately diagnose COVID‐19 patients.

## Graphical abstract

A molecular nanotechnology is developed utilizing interconnected enzyme–DNA nanostructures to achieve cooperative multi‐enzyme catalysis. Through programmable nanostructures and favorable equilibrium coupling in the system architecture, the technology achieves sensitive and versatile detection for the direct measurement of SARS‐CoV‐2 RNA targets in native patient specimens (30 min at room temperature).

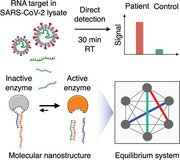

## 1 Introduction

COVID‐19 has caused a rapidly evolving pandemic.^[^ ^1^, ^2^, ^3^ ^]^ Increased testing for SARS‐CoV‐2, the causal pathogen, and its emerging variants^[^ ^4^ ^]^ is key to limiting the spread. In particular, accessible and adaptable testing that can accurately diagnose and be performed near patients (e.g., community clinics and doctors’ offices)^[^ ^5^, ^6^ ^]^ are urgently needed for responsive management. Nucleic acid testing can effectively distinguish the novel coronavirus from other pathogens, but current assays rely primarily on target amplification—target sequences need to be extensively replicated before detection—and thus pose challenges for versatile assay prototyping. For example, quantitative reverse transcription–polymerase chain reaction (RT–qPCR) are operationally complex (e.g., require thermal‐cycling and lengthy processing for target amplification)^[^ ^7^, ^8^ ^]^ and lack the programmability to adapt to new pathogen variants. Advanced isothermal amplification assays, such as loop‐mediated isothermal amplification (LAMP)^[^ ^9^, ^10^ ^]^ and clustered regularly interspaced short palindromic repeats‐based detection,^[^ ^11^, ^12^, ^13^, ^14^ ^]^ can overcome the need for temperature cycling; however, these technologies too entail exquisite primer design and face challenges for new assay design. For example, LAMP requires multiple sets of compatible primer pairs that span long genomic regions; extensive experimental optimization is thus required for new primer selection and compatibility validation to prevent false positives arising from primer dimers.

Dynamic DNA nanotechnology offers an attractive approach to enable accessible and adaptable detection.^[^ ^15^, ^16^, ^17^, ^18^ ^]^ Molecular DNA nanostructures are highly programmable to execute different functions; as modular elements, they can be designed to respond directly to specific targets and activate catalytic reactions.^[^ ^19^, ^20^ ^]^ Interestingly, such target‐induced catalysis mimics the mechanism of cellular signaling. During biological signal transduction, the specific binding of a target to its receptor triggers receptor conformational changes; this receptor activation rapidly propagates and switches cascading enzymatic reactions—typically through a series of interconnected and coordinated catalytic networks—to markedly amplify signal and reduce noise, so as to direct pivotal cellular communication and processes.^[^ ^21^, ^22^, ^23^ ^]^ Inspired by this mechanistic similarity and the extraordinary functionality of dynamic biological networks, we reason that molecular nanostructures can be programmed and organized as a synergistic network architecture, to directly detect nucleic acid targets and dramatically amplify signaling responses, thereby obviating the need for target amplification as used in traditional nucleic acid testing.

Here we describe a collaborative system of interacting molecular nanostructures that enables direct and programmable detection of SARS‐CoV‐2 RNA targets in patient specimens. Named synergistic coupling of responsive equilibrium in enzymatic network (SCREEN), the technology leverages two dynamic networks—each governed by a responsive, catalytic molecular nanostructure—to construct an interconnected system architecture. In the target recognition network, a programmable enzyme–DNA nanostructure is readily activated upon target hybridization to liberate strong enzyme activity; unlike conventional technologies that require primer pairs to span the new target sequence, the SCREEN nanostructure can be readily programmed by adapting a single short sequence to confer new target specificity. In the signal amplification network, the target‐induced enzyme activation toggles another catalytic DNAzyme nanostructure to dramatically enhance the system response.

Motivated by the simultaneous and interconnected establishment of multiple equilibriums within the system, we develop a computational model to collaboratively couple these intra‐ and inter‐network interactions. The SCREEN architecture thus mimics the organization and functionality of cellular signaling cascades. Through programmable modular nanostructures, SCREEN recognizes different RNA targets of interest; through coordinated system organization, it benefits from not only multi‐enzyme catalysis that is naturally aligned, but also favorable equilibrium coupling to fully drive the dynamic architecture, so as to achieve an enhanced system response that is larger in magnitude and faster in reaction kinetics. The developed platform efficiently converts a single RNA target binding into an amplified electrical signal, and demonstrates fast and direct detection. The SCREEN assay reaction thus bypasses conventional target amplification and is completed within 30 min at room temperature. Importantly, the platform achieves robust programmability for the integrated detection of diverse targets (across different genetic loci of SARS‐CoV‐2). When applied for clinical COVID‐19 diagnostics, SCREEN demonstrated sensitive and direct detection in clinical swab lysates to accurately diagnose infected patients.

## 2 Results

### 2.1 The SCREEN Platform

**Figure 1:**
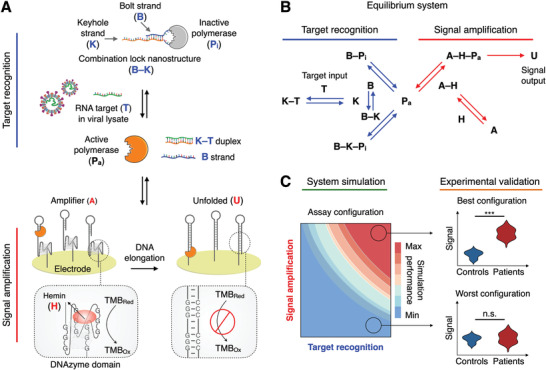
The SCREEN platform. A) Working principle of the SCREEN platform. SCREEN leverages the favorable coupling of two catalytic networks: the target recognition network and the signal amplification network. The target recognition network consists of a programmable DNA lock nanostructure (B–K) which binds and inactivates a DNA polymerase (P_i_). The lock nanostructure comprises a bolt strand (B) and a keyhole strand (K) that is designed to be complementary to the target RNA (T). In the presence of the target, the keyhole strand hybridizes with the target, displacing the bolt strand; this displacement destabilizes the lock nanostructure and releases an active polymerase (P_a_). Polymerase activation is next measured through the signal amplification network, which consists of amplifier DNA nanostructures (A) immobilized on the surface of a screen‐printed electrode. Each amplifier nanostructure comprises an upstream polymerase‐binding domain, a hairpin structure that primes any polymerase activity, and a downstream DNAzyme peroxidase domain, assembled through G‐quadruplex coupling with hemin (H). As an active polymerase binds and elongates the amplifier nanostructure, the polymerase disrupts the latter's G‐quadruplex peroxidase domain. The unfolded amplifier (U) thus loses its catalytic activity for redox reactions and produces a large resultant signal change. B) SCREEN network system. SCREEN leverages collaborative equilibrium coupling across multiple interactions within the network architecture to efficiently drive the dual‐enzyme catalysis, thereby transducing even sparse target input (T) into an amplified and rapid signal output (U). C) To establish favorable equilibrium shifts, we developed a computational model that simulated all reactions in the network system to tune molecular components, predict overall assay performance and establish equilibrium‐driven assay configurations. The simulation‐derived assay configurations were experimentally validated and clinically evaluated for COVID‐19 detection. n.s., not significant. *** indicates statistical significance.

SCREEN leverages the collaborative coupling of two catalytic networks to achieve direct and programmable detection of SARS‐CoV‐2 RNA targets in cellular lysate (**Figure** **1**A). The two networks—the target recognition network and the signal amplification network—are fully integrated to transduce a specific nucleic acid hybridization event into an amplified electrochemical signal. Specifically, the target recognition network consists of a programmable hybrid nanostructure that functions as a molecular combination lock (B–K) (Figure S1A,B, Supporting Information). In the absence of specific nucleic acid target (T) (e.g., SARS‐CoV‐2 RNA), the combination lock, comprising two synthetic oligonucleotides (the bolt B and keyhole K strand), binds to and inactivates a DNA polymerase (P_i_). Only in the presence of specific target, the keyhole strand preferentially hybridizes with the target, releasing the bolt strand and fully activating the DNA polymerase (P_a_) (Figure S1C,D, Supporting Information). By adapting the sequence of the high selectivity region in the programmable combination lock—the duplex region—the structure can be readily reprogrammed to specifically recognize different targets of interest (Figure S2, Supporting Information). This switching of polymerase activity directly interfaces with the signal amplification network that features an amplifier DNA nanostructure (A). The amplifier comprises two distinct domains: an upstream polymerase‐binding domain and a downstream DNAzyme peroxidase domain. In the absence of active polymerase, the amplifier harbors strong peroxidase activity, through its folding into a secondary G‐quadruplex structure that couples with hemin (H) to catalyze redox reactions^[^ ^24^, ^25^ ^]^ (Figure S3A,B, Supporting Information). Upon the binding of an active polymerase (i.e., to the amplifier's polymerase‐binding domain), the self‐primed amplifier is elongated, unfolding its G‐quadruplex domain (U) and destroying the catalytic peroxidase activity (Figure S3C,D, Supporting Information). SCREEN thus enables dual‐enzyme catalysis (i.e., polymerase and DNAzyme peroxidase) to achieve strong signal transduction in a one‐pot reaction. To enable robust measurement, the amplifier nanostructures are uniformly immobilized on an electrode surface (Figure S3E,F, Supporting Information); once disrupted by active polymerases, they induce an amplified change to the system's peroxidase activity and achieve strong electrochemical signaling (Figure S4, Supporting Information).

In addition to this dual‐enzyme catalysis, SCREEN leverages collaborative equilibrium coupling in the catalytic network system to achieve superior performance (Figure 1B). With the simultaneous establishment of multiple equilibriums in the SCREEN system (i.e., intra‐network and inter‐network), we reason that by adjusting its molecular compositions, we can employ favorable equilibrium shifts to further drive the dual‐enzyme amplification, thus achieving an enhanced system response that is not only larger in magnitude but also faster in kinetics. Notably, as each molecular component is interconnected with every other component through a different and yet dependent reaction chain, we established a computational model to simulate the SCREEN system (Figure 1C); the model not only allows tuning of individual components, but also enables the perturbation effects to propagate upstream and downstream, within and across the networks, thereby enabling accurate prediction of overall system response (i.e., assay performance). Through simulation‐guided and equilibrium‐driven integration, we programmed different combination locks to detect various pathogen targets, and optimized the SCREEN network configurations to achieve an ideal balance of performance metrics (i.e., signal at a low target amount (Signal_L_) and at a high target amount (Signal_H_), and speed to reach system equilibrium). We further experimentally evaluated these SCREEN assays, and clinically validated their performance in patient specimens.

### 2.2 Characterization of Network Components

**Figure 2:**
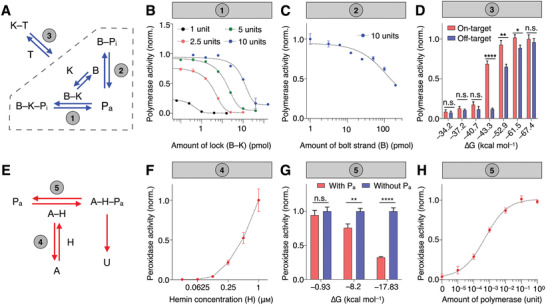
Characterization of interaction network. A) Experimental characterization of individual interactions in the target recognition network. B) Equilibrium between pre‐assembled lock nanostructure and polymerase (1). Different amounts of polymerase were subjected to a titration of lock nanostructure (B–K) and the resultant polymerase activity was measured. C) Equilibrium between bolt strand and polymerase (2). A fixed amount of polymerase was mixed with varying amounts of bolt strand (B) only and the resultant polymerase activity was measured. Without the keyhole strand, the bolt strand exhibited weak polymerase inhibition. D) Equilibrium between target and a complex mixture of lock nanostructure (3). The equilibrium shifting is affected by the target‐binding affinity (∆*G*) of the keyhole strand, resulting in varying amounts of polymerase activation to on‐target and off‐target sequences. E) Experimental characterization of individual interactions in the signal amplification network. The amplifier nanostructure consists of two domains, an upstream polymerase‐binding domain, and a downstream DNAzyme peroxidase domain. F) Equilibrium between the amplifier DNAzyme domain and hemin (4). A fixed amount of amplifier was incubated with varying amounts of hemin, to facilitate the assembly of DNAzyme peroxidase complex (A–H). The resultant peroxidase activity was measured. G) Equilibrium between the amplifier polymerase‐binding domain and polymerase (5). We varied the priming capability (∆*G*) of the amplifier's polymerase‐binding domain. Upon incubation with polymerase, which elongates the amplifier to disrupt the downstream DNAzyme peroxidase domain, we measured the resultant peroxidase activity. In the presence of a fixed amount of polymerase, the resultant peroxidase activity decreased with increasing priming capability of the polymerase‐binding domain. H) Performance of the signal amplification network. The signaling network enabled sensitive measurement of polymerase across a large dynamic range. All measurements were performed in triplicate and the data are presented as mean± s.d. (*P < 0.05, **P < 0.005, ****P < 0.0001, n.s., not significant, Student's *t*‐test).

To develop the SCREEN platform, we first evaluated individual molecular interactions in the recognition and signaling network, respectively. For every interaction, we not only verified the proposed mechanism (i.e., binding partners and expected response), but also experimentally characterized its equilibrium and kinetic properties. The recognition network can be resolved as three major reactions (**Figure** **2**A): 1) inhibition of polymerase activity by the fully‐formed combination lock (B–K); 2) inhibition of polymerase activity by the partially‐formed lock (bolt strand only, B); and 3) target‐induced polymerase activation, in a complex mixture of fully‐formed and partially‐formed locks (dotted box). Using a molecular combination lock designed to recognize the spike (S) gene of SARS‐CoV‐2 (Table S1, Supporting Information), we assessed these respective interactions. First, to a fixed amount of polymerase, we incubated an increasing concentration of pre‐assembled combination locks (B–K) (Figure 2B). The results not only confirmed the potent inhibitory effect of the fully‐formed combination lock on polymerase activity, but also demonstrated that the relative ratio between the two (combination lock:polymerase) could determine the equilibrium state of the mixture (i.e., as characterized through the resultant polymerase activity). Next, to a fixed amount of polymerase, we incubated an increasing concentration of bolt strand (B) only (Figure 2C). Interestingly, the bolt strand alone showed a weak inhibitory effect on polymerase activity. To establish the interconnectivity of lock dynamics (dotted box), by varying the relative amounts of lock constituents, we prepared complex mixtures of fully‐formed and partially‐formed combination locks (Figure S5A, Supporting Information). In the absence of target, these mixtures demonstrated different polymerase activity (P_a_), reflecting their respective initial equilibrium states; when treated with an equal amount of target, these lock mixtures experienced various target‐induced equilibrium shifts and showed different amounts of resultant polymerase activation (Figure S5B–D, Supporting Information).

We next evaluated target interaction with the complex lock mixture. In particular, we prepared molecular locks with varying target affinity (Gibbs free energy for target hybridization, ∆*G*) by changing only the overhang portion of the keyhole strand (K) (Figure S6A, Supporting Information); this strategy preserved the lock dynamics in the absence of target, thereby enabling the independent assessment of target interaction. We incubated these locks with on‐target and off‐target sequences, respectively. Their different target‐binding affinities affected the extent of equilibrium shifting, leading to different responses (i.e., on‐target polymerase activation versus off‐target activation) (Figure 2D). For example, with low‐affinity locks (∆*G* = −37.2 kcal mol^−1^), target hybridization could not perturb the lock equilibrium; both on‐target and off‐target binding resulted in negligible polymerase activation. With high‐affinity locks (∆*G* = −67.4 kcal mol^−1^), both on‐target and off‐target binding readily shifted the lock equilibrium to result in equivalent and substantial polymerase activation. An optimized lock affinity (∆*G* = −43.3 kcal mol^−1^) achieved large on‐target activation but negligible off‐target activation (Figure S6B–D, Supporting Information).

We finally assessed the signal amplification network. With the optimized design of the amplifier nanostructure (Figure S3, Supporting Information), the signaling network contains two interaction equilibriums (Figure 2E): 4) DNAzyme assembly by its constituents (i.e., G‐quadruplex DNA and hemin); and 5) polymerase interaction with the amplifier (i.e., the polymerase‐binding domain) that leads to DNAzyme disruption and loss of peroxidase activity. To characterize the DNAzyme assembly, to a fixed concentration of amplifier G‐quadruplex structure, we incubated varying amounts of hemin and measured the resultant peroxidase activity (Figure 2F). While hemin exhibited a weak background activity, its coupling with G‐quadruplex amplifier gives rise to strong DNAzyme peroxidase activity (Figure S7, Supporting Information). Next, to evaluate polymerase interaction with the amplifier (i.e., the polymerase‐binding domain, through which polymerase initiates DNA elongation), we independently varied the priming capability of the amplifier by changing its duplex 3′‐end (∆*G*, Figure S8A, Supporting Information). In the absence of polymerase binding, this change did not affect the DNAzyme peroxidase activity (Figure S8B, Supporting Information); however, when incubated with a fixed amount of polymerase, the resultant DNAzyme activity decreased proportionally with increasing amplifier priming capability, indicating a polymerase‐specific disruption of DNAzyme (Figure 2G). We finally incubated the amplifiers with different amounts of polymerase to characterize their effects on the disruption of DNAzyme activity (Figure 2H). Our results showed that this disruption could be used as a sensitive measure of the amount of active polymerase.

### 2.3 Equilibrium‐Driven Assay Configuration

**Figure 3:**
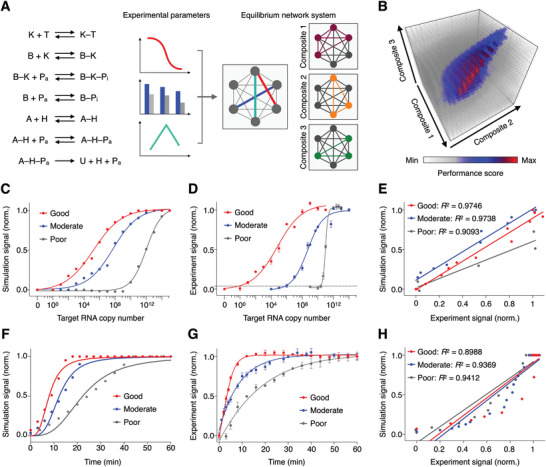
Simulation‐based assay configuration. A) Computational model of the SCREEN network architecture. Using experimental equilibrium and kinetic properties measured of individual network reactions, we developed the model to simulate all reactions as chained equations to reflect and optimize coupling relationships between and among reactions. By component connectivity, the SCREEN system could be resolved as three major reaction composites. The model was performed with a varying amount of target, over a series of composite combinations, to optimize assay configuration for enhanced performance. B) Simulation result. Different assay configurations were simulated and presented as a performance score, which integrated key performance parameters, signals at high and low concentration of target, and speed to reach system equilibrium, using a weighted scoring system. C–E) The optimized assay configuration demonstrated enhanced signal. C) Simulated and D) experimental titration curves of representative good, moderate and poor performance assays as determined by the SCREEN model. The dotted line indicates the experimentally‐determined detection limit of the good performance assay configuration. The detection limit is defined as 3 × s.d. above the signal of the no‐target sample. Signals above this detection limit are considered distinguishable from the blank with >99% confidence. E) Correlation between experimental and simulated signals of the good, moderate and poor performance assays. F–H) The optimized assay configuration demonstrated enhanced kinetics. F) Simulated and G) experimental real‐time signal generated in response to target by representative good, moderate and poor performance assays as determined by the SCREEN model. H) Correlation between experimental and simulated signals of the good, moderate and poor performance assays. All experimental measurements were performed in triplicate and the data are presented as mean± s.d.

Motivated by the simultaneous and interconnected establishment of multiple equilibriums in the SCREEN platform, we reason that these intra‐ and inter‐network interactions could be readily coupled;^[^ ^26^, ^27^ ^]^ through collaborative equilibrium coupling, target‐induced system response (i.e., U) could be further driven by favorable equilibrium shifts and non‐linearly enhanced to fully exploit the dual‐enzyme catalysis. To achieve an optimal assay configuration that maximizes this equilibrium‐driven amplification, we developed an integrated model based on both experimental characterization and computational simulation to establish the SCREEN network architecture and evaluate different combinations of reaction conditions (**Figure** **3**A and Figure S9, Supporting Information). Specifically, the model utilizes experimentally‐determined equilibrium and kinetic constants of individual network reactions (Figure S10, Supporting Information) to computationally regulate a network of corresponding reactions (Figure S11, Supporting Information). The integrated model not only allows tuning of individual molecular components, but also predicts overall assay performance. We thus used this model to simulate the effects of various assay configurations, and predict their overall assay performance with respect to three key evaluation parameters (Figure S12, Supporting Information). For example, in an assay configuration designed to enhance signal intensity, through favorable equilibrium shifting, the simulated response increases non‐linearly with target amount (Figure S13A, Supporting Information). We empirically constructed this assay configuration and verified that the experimental results agreed well with that of the simulation (Figure S13B, Supporting Information).

For SARS‐CoV‐2 detection, we next formulated a weighted scoring system based on, in order of priority, Signal_L_, Speed, and Signal_H_ performance metrics to assess and rank different assay configurations (Figure 3B). To validate the fidelity of the SCREEN computation model, we selected three assay configurations representative of different performance scores (i.e., good, moderate, and poor) and experimentally evaluated these configurations. In agreement with the simulation results, the best assay configuration achieved an enhanced system response that is not only larger in magnitude but also faster in kinetics. In terms of magnitude amplification, the good configuration demonstrated the best limit of detection (LOD = single copy v.s. 10^6^ copies v.s. 10^10^ copies) and dynamic range (10 orders v.s. 10 orders v.s. 4 orders), when compared to the moderate and poor configurations (Figure 3C,D and Figure S13C, Supporting Information). The experimental results correlated well with the predicted results (good: *R* ^2^ = 0.9746; moderate: *R* ^2^ = 0.9738; poor: *R* ^2^ = 0.9093, Figure 3E). We attribute this superior performance to the SCREEN's dual‐enzyme catalysis and collaborative equilibrium coupling in this interconnected system (Figure S13D, Supporting Information). In terms of reaction kinetics, the good configuration was also the fastest, with the reaction completing within 15 min, as compared to 30 and 60 min of the moderate and poor configurations, respectively (Figure 3F,G). The experimental kinetics correlated well with the simulated results (good: *R* ^2^ = 0.8988; moderate: *R* ^2^ = 0.9369; poor: *R* ^2^ = 0.9412, Figure 3H). Furthermore, the good configuration also demonstrated the largest on‐target signal and the lowest off‐target background signal (Figure S14, Supporting Information).

### 2.4 Programmable Nucleic Acid Target Detection

**Figure 4:**
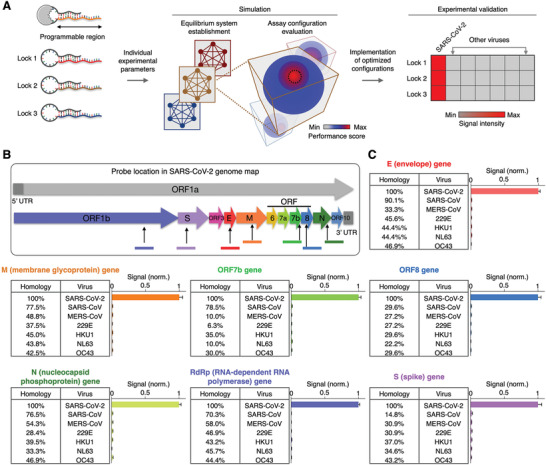
Programmable SCREEN assays for different targets. A) New SCREEN assay development. The lock nanostructure contains a programmable region that can be varied at will to detect different targets of interest. For these new lock designs, we experimentally characterize their respective equilibrium and kinetic parameters, and employ the SCREEN computation model to establish and evaluate the performance of millions of assay configurations. Optimized SCREEN assays are selected from the simulation study and experimentally validated. B) SARS‐CoV‐2 genome map. Gene sections of interest used in this study are indicated. Not drawn to scale. C) SCREEN assays for seven different SARS‐CoV‐2 gene targets. Using the SCREEN assay development workflow, we established new assays for different SARS‐CoV‐2 gene targets and experimentally evaluated their specificity, through testing with other pathogen sequences with various levels of homology. All measurements were performed in triplicate and the data are presented as mean± s.d.

We next applied the SCREEN model to develop other molecular combination locks and establish assay configurations for various nucleic acid targets of interest (**Figure** **4**A). We designed new molecular locks, through incorporating different keyhole strands (Figure S15, Supporting Information). Specifically, we leveraged the strong sequence selectivity of the duplex region in the programmable lock nanostructure to design highly specific SARS‐CoV‐2 assays; key sequences of SARS‐CoV‐2 targets were designed to maximize complementary matching with the duplex region. We further characterized the reaction parameters of these new molecular locks, and applied the SCREEN computation model to exhaustively evaluate the performance of millions of assay configurations. Optimized SCREEN assays were selected from the simulation study and experimentally validated.

To evaluate the feasibility of this workflow in developing new assays, we designed molecular locks for different SARS‐CoV‐2 gene targets and assessed their performance. In addition to the spike (S) gene, we designed six other lock nanostructures for different targets in the SARS‐CoV‐2 genome, namely the envelope (E), membrane glycoprotein (M), nucleocapsid (N), open reading frame 7b (ORF7b), open reading frame 8 (ORF8) and the RNA‐dependent RNA polymerase (RdRp) genes (Figure 4B). For each of these molecular locks, we computationally optimized the assay configuration and experimentally validated the assay (Figure S15B,C, Supporting Information). To evaluate each assay's specificity, in addition to its SARS‐CoV‐2 RNA target (on‐target), we used homologous sequences from other related SARS‐like coronaviruses (off‐targets) (Figure S16, Supporting Information). When experimentally evaluated, the established SCREEN assays distinguished SARS‐CoV‐2 with little cross‐reactivity to related coronaviruses (Figure 4C) as well as other pathogens such as dengue and H1N1 virus (Figure S17, Supporting Information), and demonstrated negligible signal in the presence of human transcriptome (Figure S18, Supporting Information).

We next assessed the ability of the SCREEN platform to perform multiplexed detection in a single reaction. Simultaneous detection of multiple targets in a single test not only improves the detection sensitivity (e.g., across different genetic loci of SARS‐CoV‐2) but also enables broad‐spectrum coverage (e.g., different mutations and/or different strains). Using a combination of different molecular locks (i.e., N and S gene), we first established a dual‐target assay that could produce a strong signal when either target was present (Figure S19A, Supporting Information). We further expanded this approach to develop a three‐target configuration (i.e., N, S, and RdRp gene) to improve the detection coverage (Figure S19B, Supporting Information).

### 2.5 Detection of SARS‐CoV‐2 in Clinical Samples

**Figure 5:**
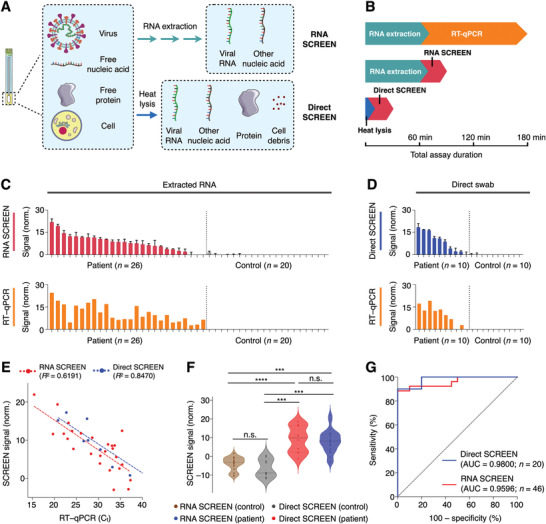
Rapid detection of SARS‐CoV‐2 in clinical samples. A) Schematic representation of different sample preparation and SCREEN assays. SCREEN can be applied to analyze extracted RNA (RNA SCREEN) as well as clinical swab lysates (direct SCREEN, bypasses RNA extraction). B) Comparison of assay duration. Gold‐standard RT–qPCR involves RNA extraction and a long assay duration, requiring at least 180 min to finish the whole procedure. Regardless of the sample type (extracted RNA v.s. swab lysate), the SCREEN procedure can be completed in 30 min at room temperature. Notably, in direct SCREEN, as no RNA extraction is required, sample preparation and assay reaction can be completed in as little as 35 min. C–D) SCREEN and RT–qPCR measurements of clinical COVID‐19 samples. Swab samples were collected from patients and controls and subjected to C) RNA SCREEN (*n* = 46; 26 patient samples and 20 control samples) or D) direct SCREEN (*n* = 20; 10 patient samples and 10 control samples). Gold‐standard RT–qPCR measurements were performed as a sample‐matched comparison and the data are presented to reflect viral RNA load. E) Correlation of SCREEN and RT–qPCR analysis. Both the RNA SCREEN and direct SCREEN showed a good agreement with the RT–qPCR analysis, even in samples with a low viral load (*C*_t_ > 35). F) Multiple comparison analysis of clinical SCREEN measurements. Regardless of the measurement approach (RNA SCREEN or direct SCREEN), patient samples produced significantly higher signals as compared to control samples. The different SCREEN assays showed no significant difference within the patient population. G) Receiver operating characteristic (ROC) curves of RNA SCREEN (*n* = 46, AUC = 0.9596) and direct SCREEN (*n* = 20, AUC = 0.9800). All measurements were performed in triplicate and the data are presented as mean± s.d. before assay thresholding in (C,D). a.u., arbitrary unit. RT–qPCR signals in (C,D) are presented as 40–*C*_t_. AUC, area under the curve. (***P < 0.0005, ****P < 0.0001, n.s., not significant, Student's *t*‐test).

We finally applied the SCREEN platform for clinical detection of SARS‐CoV‐2 in patient samples (i.e., extracted RNA and swab lysates) (**Figure** **5**A). As SCREEN detects via direct target hybridization, rather than through target amplification, we reason that the platform could have improved compatibility with complex swab lysates. To enable direct detection in nasopharyngeal swabs, we developed a lysis treatment to release viral RNA and preserve RNA integrity. We found that a short heat lysis (75 °C, 5–30 min),^[^ ^28^ ^]^ in the presence of a suitable stabilization buffer, not only released RNA but also preserved RNA integrity (Figure S20A–D, Supporting Information). We then employed the SCREEN platform to detect RNA targets directly in these swab lysates. As hypothesized, SCREEN performed robustly. It showed minimal interference against the complex lysate background and demonstrated a high correlation with gold standard RT–qPCR (Figure S20E, Supporting Information). In comparison to conventional RT–qPCR (i.e., 3‐h processing), the SCREEN assay workflows (i.e., RNA SCREEN with extracted RNA or direct SCREEN with cell lysates) are notably shorter (Figure 5B). In particular, direct analysis can be accomplished in as little as 35 min (5‐min heat lysis to prepare cell lysates and 30‐min direct SCREEN assay at room temperature); this process bypasses all steps of RT–qPCR (e.g., RNA extraction, reverse transcription, and thermal cycling‐based target amplification). To facilitate robust SCREEN implementation, we further lyophilized the assay reagents and demonstrated their stable activity, even after 3 weeks of storage at −20, 25 °C and under accelerated aging (80 °C) (Figure S20F, Supporting Information).

Using both types of clinical specimens, extracted RNA (Figure 5C), and swab lysates (Figure 5D), we evaluated the SCREEN workflow to detect SARS‐CoV‐2 in these patient samples. When correlated with sample‐matched RT–qPCR quantification, the SCREEN measurements demonstrated a high accuracy and comparable sensitivity, even in samples with a low viral load (*C*_t_ > 35) (RNA: *R* ^2^ = 0.6191; swab: *R* ^2^ = 0.8470, Figure 5E). To further assess the SCREEN assays in different sample types, we performed multi‐comparison analyses in positive and negative samples, respectively (Figure 5F). The SCREEN measurements of extracted RNA samples and direct swab samples were comparable within the control and patient population, respectively. For inter‐population comparison, SCREEN analyses of the patient population were significantly higher than that of the control population for both sample types. These results indicate that the direct lysis approach is comparable in performance to the standard RNA extraction method to release and preserve RNA from clinical swabs. In comparison to the gold standard RT–qPCR test, our optimized SARS‐CoV‐2 SCREEN assay demonstrated a high accuracy in distinguishing infected patients from control samples (extracted RNA: area under the curve (AUC) = 0.9596, swab lysates: AUC = 0.9800, Figure 5G).

## 3 Discussion

The ongoing COVID‐19 pandemic highlights the critical need for accessible and adaptable testing, particularly in response to a rapidly evolving pathogen.^[^ ^4^, ^29^ ^]^ Current nucleic acid detection technologies, however, face challenges in achieving these goals. Based on the conventional approach of target amplification, current methods rely primarily on primer pairs and distinct enzymes to define and amplify RNA targets of SARS‐CoV‐2, through thermal cycling or isothermal processes, before detection. With respect to assay implementation, these tests thus require complex sample processing and extensive assay development, to accommodate the stringent requirements by different enzymes and sequences; with respect to assay versatility, these approaches entail exquisite primer design and become increasingly complex for multiplexed integration and/or new assay prototyping (e.g., new sets of compatible primer pairs).^[^ ^7^, ^9^, ^11^, ^12^ ^]^ Motivated by the direct activation and enhanced response of biological signaling pathways, we developed the SCREEN platform to address these challenges. In comparison to existing SARS‐CoV‐2 nucleic acid detection technologies (Table S3, Supporting Information), SCREEN offers an integrated molecular nanotechnology to achieve direct and programmable diagnostics. The technology leverages catalytic molecular nanostructures to establish a collaborative architecture for multi‐enzyme catalysis. As compared to traditional DNAzymes,^[^ ^30^ ^]^ the SCREEN nanostructures are hybrid complexes comprising nucleic acids and protein enzymes; these nanostructures are not only responsive to nucleic acid targets but also potent catalysts, due to their high‐efficiency constituent protein‐enzymes. Notably, SCREEN benefits from the collaborative coupling of these hybrid nanostructures to dramatically enhance its system output, thereby enabling accessible implementation and versatile programmability.

In terms of accessible implementation, SCREEN is well‐suited for the direct detection of rare targets in clinical specimens: 1) SCREEN does not require target amplification. Specifically, it detects through direct target binding to activate a polymerase, and polymerase‐based elongation to disrupt a DNAzyme. Furthermore, akin to biological cascades, this assay design has minimal compatibility issues with enzymes and sequences; it utilizes enzymatic processes that are naturally aligned, upstream and downstream, along a signaling pathway to enhance the detection response. As a result, SCREEN performs robustly even against a complex biological background (e.g., cellular lysates), bypasses all processing steps of conventional methods (i.e., RNA extraction, reverse transcription, and target amplification); 2) SCREEN benefits from favorable equilibrium shifts within the system architecture to dramatically enhance its system output. Powered by this collaborative coupling of reactions, SCREEN transduces a single target binding event into a nonlinearly‐amplified electrical signal. The technology thus achieves a high sensitivity (approaching single‐copy detection) and fast response (30 min at room temperature); and 3) the detection requires minimal equipment and can be readily implemented with portable potentiostats (e.g., blood glucometers), making the technology ideal for point‐of‐care and large‐scale diagnostics.

In terms of assay programmability, SCREEN's assay architecture—modular nanostructures and integrated system—enhances new assay prototyping. As modular units, new molecular nanostructures can be readily designed to achieve precise specificity, against new targets of interest. As an interconnected network system, SCREEN employs computational modeling as a universal platform to guide the incorporation of new molecular nanostructures (and their combinations for multiplexed recognition), evaluates overall assay performance, and establishes equilibrium‐driven assay configurations. With the emergence of strain mutations in SARS‐CoV‐2^[^ ^4^ ^]^ and other novel infectious agents,^[^ ^31^ ^]^ we anticipate that the SCREEN platform could be readily programmed to detect new pathogens and/or mutations; its versatile programmability and facile integration enable rapid assay prototyping and implementation to expedite emergency disease control. The technology has the potential to be expanded further. To achieve additional catalytic coupling, new reactions involving other catalysts,^[^ ^32^, ^33^ ^]^ DNAzymes,^[^ ^34^ ^]^ and hybrid molecular nanostructures^[^ ^35^, ^36^ ^]^ could be incorporated to not only enhance the system response, but also expand its readout capabilities (e.g., fluorescence and colorimetry).^[^ ^37^ ^]^ To advance the network architecture, more sophisticated system designs could be integrated. For example, as compared to the current open‐nonlinear system architecture, technical improvements using closed‐loop systems^[^ ^38^ ^]^ are likely to further enhance the assay performance and enable advanced functionalities of biological dynamic networks (e.g., feedback control and noise reduction).^[^ ^39^, ^40^ ^]^ Clinically, we further anticipate that the SCREEN platform can be readily expanded to detect multiple biomarkers. With its demonstrated robustness in patient specimens, SCREEN could be applied to various clinical samples (e.g., blood, urine),^[^ ^41^, ^42^ ^]^ across a spectrum of diseases (e.g., other infectious diseases, cancers). Further microfluidic integration^[^ ^43^, ^44^ ^]^ as well as array‐type sensor implementation^[^ ^45^ ^]^ could improve the detection throughput for adaptable diagnostics near patients.

## 4 Experimental Section

### Lock Nanostructure Characterization

All oligonucleotide sequences can be found in Tables S1 and S2, Supporting Information and were purchased from Integrated DNA Technologies (IDT). Stock solutions of oligonucleotide sequences (100 µm) were prepared in Tris–EDTA buffer (pH 8.0) and stored at −20 °C, before being diluted to the required concentrations. For lock nanostructure preparation, different concentrations of oligonucleotide components (bolt strand and keyhole strand) were incubated at 95 °C for 5 min in a reaction buffer of 50 mm NaCl, 1.5 mm MgCl_2,_ and 50 mm Tris–HCl (pH 8.5). The mixture was then slowly cooled to room temperature at 0.1 °C s^−1^ and Taq DNA polymerase (Promega) was added to form the lock nanostructure. To characterize the response of different lock nanostructures to nucleic acid targets, synthetic oligonucleotides were used as matching target sequences. Titrations of target sequences were added to the lock nanostructure solution and the resultant polymerase activity was measured through 5′ exonuclease degradation of a fluorescent probe. Briefly, fluorescent probe, template, and primer (IDT) were mixed at equal concentrations with deoxynucleotide triphosphates (dNTPs, Thermo Scientific) in a reaction buffer of 50 mm NaCl, 1.5 mm MgCl_2_, and 50 mm Tris–HCl (pH 8.5). Lock nanostructures, with varying amounts of target sequences, were added to the probe mixture and incubated at 25 °C while fluorescence readings were measured (Figures S1C and S13D, Supporting Information).

### Amplifier Nanostructure Characterization

Amplifier oligonucleotide mixture was prepared by mixing self‐primed G‐quadruplex DNA structures (IDT) with dNTPs (Thermo Scientific) and 6‐carboxyfluorescein (6‐FAM, Sigma) in a buffer of 50 mm NaCl, 1.5 mm MgCl_2_, 0.1 m KCl, and 50 mm Tris–HCl (pH 8.5). The mixture was heated to 95 °C for 5 min, cooled slowly to room temperature, and mixed with hemin solution (Sigma). Sample was mixed with the amplifier oligonucleotide mixture and incubated for 30 min at room temperature before addition of equivolume amount of QuantaRed chemifluorescence substrate (Thermo Scientific). Fluorescence intensity was then measured according to manufacturer's recommendation with a microplate reader (Tecan). For each sample, the fluorescence intensity of 6‐FAM was concurrently measured for signal normalization.

### Synthetic RNA Synthesis

Target sequences were designed with a T7 promoter sequence (TAATACGACTCACTATAGG) upstream. The oligonucleotide and its reverse complement sequence were annealed by heating at 95 °C for 5 min in a buffer of 50 mm NaCl, 1.5 mm MgCl_2,_ and 50 mm Tris–HCl (pH 8.5) and slowly cooling to room temperature at 0.1 °C s^−1^. The annealed template was incubated with T7 RNA polymerase (NEB) at 37 °C for 2 h. RNase‐free DNase I (Promega) was then added to the reaction and incubated at 41 °C for 20 min. A final concentration of 0.8 m LiCl was added to the solution and the mixture was incubated at −20 °C for 2 h. The precipitate was collected by centrifuging at 16 000 g for 15 min at 4 °C, and the pellet was rinsed with freshly‐prepared 70% ethanol three times. The final pellet was air‐dried for 15 min at room temperature before being redissolved in nuclease‐free water. The quality and quantity of extracted RNA were measured with a spectrophotometer (Thermo Scientific) and stored at −80 °C before being used.

### Equilibrium and Kinetic Characterization

To model the integrated SCREEN system as a series of reversible equilibrium reactions with common molecular components, the equilibrium and kinetic parameters of each reaction were determined first. Experimental measurements of the lock nanostructure provided characterization data for reactions involving polymerase, bolt strand, keyhole strand, and target strand interactions. Experimental measurements of the amplifier nanostructure provided characterization data for reactions involving polymerase, hemin, and amplifier strand interactions. The equilibrium and kinetic properties of the SCREEN system designed to recognize the S gene of SARS‐CoV‐2 are summarized in Figure S10, Supporting Information. For oligonucleotide hybridization (reactions 1 and 2), equilibrium constants at 25 °C were determined using the van't Hoff equation. For all other reactions, equilibrium constants were determined experimentally by calculating the reaction quotient at equilibrium. To determine the kinetic constants, real‐time binding measurements were performed using biolayer interferometry (Pall ForteBio). Changes in optical thickness of the biolayer were measured as wavelength shifts in a continuous manner and the curve was fitted to determine the binding kinetics. To evaluate the interaction between amplifier oligonucleotides and hemin (reaction 5), the rate constant was determined by measuring the initial rate of the reaction. Briefly, amplifier oligonucleotides and hemin were mixed and allowed to incubate for varying durations. QuantaRed chemifluorescence substrate (Thermo Scientific) was then added and the fluorescence intensity was measured as previously described.

### SCREEN Simulation

Using these equilibrium and kinetic parameters, a two‐stage iterative simulation was established to model the experimental workflow of lock nanostructure preparation and target incubation with this mixture for signal readout. Simulation architecture is found in Figure S11, Supporting Information. In the first stage, the simulation representative of the recognition network was initialized, as characterized by the corresponding reaction parameters (*ϴ_2_*, *ϴ_3_*, and *ϴ_4_*). This was performed in the absence of target, by inputting concentrations of the three molecular lock constituents ([B]^initial^, [K]^initial^, and [P_a_]^initial^). The model simultaneously calculated the changes in concentration of different reaction components ([B], [K], [P_a_], [B–K], [B–P_i_] and [B–K–P_i_]). For cycle propagation, the computed concentrations from the previous cycle were used as inputs for the current cycle. The simulation was iterated (>1000 cycles) to equilibrate all component concentrations. In the second stage, these steady‐state concentration outputs from the first‐stage simulation (e.g., [K]^eqm^, [B]^eqm^) were used, in the presence of targets and signaling constituents ([T]^initial^, [A]^initial^, and [H]^initial^), to initialize an expanded network of reactions. The expanded network reflected both target recognition reactions and signal amplification reactions (*ϴ_1_*–*ϴ_6_*) and computed changes in concentration of the reaction components ([B], [K], [P_a_], [B–K], [B–P_i_], [B–K–P_i_], [K–T], [T], [A], [H], [A–H] and [A–H–P_a_]). By varying the input concentration of target ([T]^initial^), the concentration of output ([A–H–P_a_]^final^) was determined to evaluate the assay performance based on three parameters (i.e., Signal_L_, Speed, and Signal_H_). To facilitate assay optimization for point‐of‐care SARS‐CoV‐2 detection, these three performance parameters were combined into a single performance score by a multiplicative weighted scoring system, prioritizing Signal_L_, Speed, and Signal_H_.

### SCREEN Experimental Workflow

To implement the optimized assay configuration, as determined by the computational model, the molecular nanostructures were prepared accordingly. Using the good assay configuration designed for the S gene of SARS‐CoV‐2 as an example, the lock nanostructure was assembled from DNA constituents mixed in an optimized ratio (final concentration of keyhole strand, 0.6 µm and bolt strand, 0.6 µm). To prepare the amplifier nanostructures, thiol‐modified G‐quadruplex DNA (10 µm) was incubated with screen‐printed electrodes (SPE, Metrohm). These disposable screen‐printed electrodes have a 4‐mm diameter gold working electrode, with gold counter and silver pseudo‐reference electrodes. All incubation volumes were kept to 50 µL. After an overnight incubation, the electrodes were rinsed with excess phosphate‐buffered saline (PBS) to remove the unbound DNA, and incubated with hemin solution (100 µm) for 10 min at room temperature to fold the immobilized amplifier DNA into functional DNAzyme peroxidase. The prepared lock mixtures and functionalized electrodes could be stored until assay utilization.

To perform the SCREEN assay, all reaction volumes were kept to 50 µL. For every measurement, before sample incubation, a baseline electrochemical reading was first performed, so as to normalize for variations in electrode properties. Briefly, the electrode was rinsed with excess PBS and its amplifier‐induced enzymatic activity was measured by adding an optimized concentration of 3,3′,5,5′‐tetramethylbenzidine (1 × TMB, Sigma T4444, 50 µL) as the peroxidase substrate. Next, to perform sample incubation, the electrode was rinsed with excess PBS and it was incubated with the reaction mixture, so as to enable polymerase activation and reaction with the amplifier nanostructures. The reaction mixture was prepared by mixing a sample containing varying amounts of RNA targets with the prepared lock mixture to form a 50‐µL reaction. The incubation was maintained for 15 min at room temperature. Subsequently, the electrode was rinsed with excess PBS, before being incubated with TMB again (as described above), to measure its resultant enzymatic activity. The whole assay procedure was completed within 30 min at room temperature. For every sample measurement with the target‐specific lock nanostructure, a sample‐matched control was performed by mixing the sample aliquot with a scrambled control lock nanostructure. All electrochemical measurements were performed via chronoamperometry (Metrohm), by stepping the potential from open circuit potential (OCP) to +0.05 mV versus Ag pseudo‐reference electrode, where TMB underwent reduction, until a steady‐state current was achieved (≈180 s). See Figure S4B,C, Supporting Information for examples of the electrochemical current measurement, before and after sample incubation.

### Electrochemical Analysis

The following equations were used to determine the SCREEN signal.

(1) $$\tilde{I}=I_{\mathrm{before}}-I_{\mathrm{after}}$$

where $\tilde{I}$ indicates the current difference between the baseline measurement before sample incubation (*I*_before_) and the measurement after sample incubation (*I*_after_).

(2) $$\mathrm{SCREEN}\;\mathrm{signal}={\tilde{I}}_{\mathrm{target}}-{\tilde{I}}_{\mathrm{control}}$$

where $\tilde{I}$_target_ indicates the electrochemical measurement associated with a lock nanostructure recognizing a specific target sequence, and $\tilde{I}$_control_ indicates the measurement of a sample‐matched control (i.e., sample incubated with scrambled lock nanostructure).

### SARS‐CoV‐2 Target Selection and Lock Nanostructure Design

Genome sequences of SARS‐CoV‐2 (NC_045512), SARS (FJ882957), MERS (NC_019843), 229E (MF542265), HKU1 (MH940245), NL63 (MG772808), OC43 (AY391777), dengue virus (NC_001477), and influenza A subtype H1N1 virus (strain A/California/07/2009(H1N1), NC_026431–NC_026438) were obtained from NCBI RefSeq. Multiple sequence alignment was performed using the UGENE suite of tools.^[^ ^46^ ^]^ Regions within different genes were selected based on variability within the virus family and sequence. Highly conserved regions (>95%) were not selected as candidates to minimize chances of designing non‐specific lock nanostructures. Regions with high GC content (>75%) and repetitive regions were similarly eliminated to minimize mishybridization and secondary structure artifacts. Off‐target sequences were designed by selecting sequences in the multiple sequence alignment which aligned to the SARS‐CoV‐2 regions of interest. For influenza and dengue, sequences in different genes that had the highest similarity with the SARS‐CoV‐2 regions of interest were chosen.

### Multiplexed Target Detection

Lock nanostructures designed against respective SARS‐CoV‐2 targets (i.e., E, M, and N genes) were prepared and characterized individually to validate their performance. To create a high coverage SARS‐CoV‐2 detection assay that would respond to any and all the targets, the molecular components of these three validated lock nanostructures were mixed, in optimized ratios, and used as described above. This solution was mixed with samples and incubated with electrode‐functionalized amplifier nanostructure as previously described to determine the assay performance. Normalized signals above the detection threshold (i.e., >3 × s.d. of the background signal) were considered as positive signals, otherwise they were called null signals.

### Reagent Lyophilization

To facilitate SCREEN implementation, the reagent mixture containing the lock nanostructures (final concentration of keyhole strand, 0.6 µm and bolt strand, 0.6 µm) was lyophilized overnight in the reaction buffer (50 mm NaCl, 1.5 mm MgCl_2_, and 50 mm Tris–HCl, pH 8.5). The stability and performance of the lyophilized reagents were evaluated after storage for 3 weeks at −20, 25 °C, and under accelerated aging (80 °C).

### Cell Culture and Direct Lysis

Human lung epithelial cell line (PC9) was obtained from American Type Culture Collection and grown in RPMI‐1640 medium (HyClone) supplemented with 10% fetal bovine serum (FBS, HyClone) and 1% penicillin–streptomycin (Gibco) in a humidified 37 °C incubator with 5% CO_2_. The cell line was tested and free of mycoplasma contamination (MycoAlert Mycoplasma Detection Kit, Lonza, LT07‐418). For direct cell lysis, cells were resuspended in a stabilization/lysis buffer consisting of the reaction buffer (50 mm NaCl, 1.5 mm MgCl_2_, and 50 mm Tris–HCl, pH 8.5) with 0.2 U µL^−1^ RNase inhibitor (Promega) and incubated at 75 °C for 5 min. The resulting mixture was then rapidly cooled and stored at −80 °C before being used.

### RNA Extraction

Total RNA was isolated from cells using RNeasy kit (Qiagen), according to the manufacturer's protocol. 2 × 10^6^ cells were resuspended in RLT lysis buffer, and vortexed for 1 min at maximum speed. The quality and quantity of extracted RNA were measured with a spectrophotometer (Thermo Scientific) and the product was stored at −80 °C before being used.

### RT–qPCR Analysis

To detect specific RNA targets through gold‐standard RT–qPCR analysis, extracted RNA was first reverse‐transcribed to generate first‐strand cDNA (High‐Capacity cDNA Reverse Transcription Kit, Life Technologies). Reverse transcription was performed at the following condition: 25 °C for 10 min, 37 °C for 120 min, and 85 °C for 5 min. Quantitative PCR was performed using appropriate TaqMan primers for ACTB and GAPDH (Life Technologies). PCR condition consisted of 1 cycle of 20 °C for 2 min and 95 °C for 2 min, 45 cycles of 95 °C for 1 s and 60 °C for 20 s. All thermal cycling was performed on QuantStudio 5 real‐time PCR system (Applied Biosystems).

### RNA Degradation Analysis

RNaseAlert substrate (IDT) was dissolved in 10 × RNaseAlert buffer and kept on ice. Heat‐lysed cells were mixed with the substrate solution and the resulting mixture was incubated at 75 °C for 30 min and 25 °C for 60 min, and monitored for fluorescence signal at 488 nm excitation and 520 nm emission wavelengths. Signal was scaled to the inverse of the maximum fluorescence signal to determine the amount of intact RNA in the solution during the incubation.

### Clinical Samples

SARS‐CoV‐2 positive clinical samples were handled according to the Singapore Ministry of Health Biosafety Branch and the NUS Institutional Biosafety Committee regulations in the Biosafety Level 3 (BSL‐3) or Biosafety Level 2+ (BSL‐2+) laboratories where appropriate. Subjects were recruited from multiple independent cohorts using Institutional Review Board‐approved protocols. Total RNA was extracted using EZ1 Advanced XL and EZ1 Virus Mini Kit 2.0 (Qiagen). Extracted RNA was eluted in nuclease‐free water and stored at −80 °C before being used. For direct SCREEN analysis, nasopharyngeal swabs were collected in stabilization/lysis buffer. Per institutional guideline, these clinical samples were heat‐treated for 30 min at 75 °C for additional safety precaution. All clinical samples were treated at the point of sample collection, at the clinical institution, before their transfer to research laboratories. All heat‐inactivated samples were stored at −80 °C before being used.

### Clinical Measurements

For RNA SCREEN analysis of purified RNA, 5 µL of extracted RNA was used as the input material. For direct SCREEN, 35 µL of nasopharyngeal swab lysate was used. The SCREEN assays were performed at room temperature and completed within 30 min. For clinical benchmarking, SARS‐CoV‐2 clinical diagnoses were generated by commercial RT–qPCR assay (Fortitude Kit, MiRXES). Amplification conditions consisted of 1 cycle of 48 °C for 15 min, 1 cycle of 95 °C for 150 s, 42 cycles of 95 °C for 10 s and 59 °C for 42 s. All SCREEN measurements on clinical samples were performed in an anonymized and blinded fashion and finalized before comparison with clinical reports.

### Statistical Analysis

Unless otherwise stated, all measurements were performed in triplicate, and the data displayed as mean ± standard deviation. Significance tests were performed via a two‐tailed Student's *t*‐test or ANOVA. For inter‐sample comparisons, multiple pairs of samples were each tested, and the resulting *P* values were adjusted for multiple hypothesis testing using Bonferroni correction. Values that had an adjusted *P* < 0.05 were determined as significant. Receiver operating characteristic curves were generated from patient profiling data and constructed by plotting sensitivity versus (1–specificity), and the values of area under the curve (AUC) were computed using the trapezoidal rule. The clinical reports were used as classifiers (true positives and true negatives). Samples with *C*_t_ values greater than 40 for both of the SARS‐CoV‐2 targets were considered to be negative. Detection sensitivity, specificity, and accuracy were calculated using standard formulas. All modeling and analyses were performed using R (version 3.5.0) and GraphPad Prism (version 8.4.3).

## Conflict of Interest

Y.C., N.R.S., N.R.Y.H., and H.S. are inventors on a pending patent application related to this work filed by the National University of Singapore. The other authors declare no competing interests.

## Author Contributions

Y.C., N.R.S., and N.R.Y.H. contributed equally to this work. Y.C., N.R.S., N.R.Y.H., and H.S. designed the research. Y.C., N.R.S., N.R.Y.H., A.N., and Y.L. performed the research. Q.H.M. and Y.W. performed BSL‐3 experiments that allowed clinical sample testing. D.L.L.B., K.L.C., D.C., P.A.T., and C.W.M.O. provided de‐identified clinical samples and data. Y.C., N.R.S., N.R.Y.H., A.N., Y.L., and H.S. analyzed the data and wrote the paper. All authors contributed to the manuscript.

## Supporting information

Supporting Information

## Data Availability

The data that support the findings of this study are available from the corresponding author upon reasonable request.
