# Supplementary material for: Collaborative Equilibrium Coupling of Catalytic DNA Nanostructures Enables Programmable Detection of SARS‐CoV‐2
Source: Adv Sci (Weinh). 2021 Jul 18;8(18):2101155. doi: 10.1002/advs.202101155 (PMC8420304; doi:10.1002/advs.202101155)
Supplement: Supplementary file 1 — Supporting Information [file ADVS-8-2101155-s001.pdf]

© 2021 Wiley-VCH GmbH

ADVANCED SCIENCE
Open Access

Supporting Information

for *Adv. Sci.,* DOI: 10.1002/advs.202101155

Collaborative equilibrium coupling of catalytic DNA nanostructures enables programmable detection of SARS-CoV-2

Yuan Chen, Noah R. Sundah, Nicholas R.Y. Ho, Auginia Natalia, Yu Liu, Qing Hao Miow, Yu Wang, Darius L.L. Beh, Ka Lip Chew, Douglas Chan, Paul A. Tambyah, Catherine W. M. Ong, Huilin Shao*

SUPPLEMENTARY INFORMATION

Collaborative equilibrium coupling of catalytic DNA nanostructures enables programmable detection of SARS-CoV-2

Yuan Chen[1,2,#], Noah R. Sundah[1,2,#], Nicholas R.Y. Ho[1,3,#], Auginia Natalia[1,2], Yu Liu[1,2], Qing Hao Miow[4], Yu Wang[4], Darius L.L. Beh[5], Ka Lip Chew[6], Douglas Chan[7], Paul A. Tambyah[4,5], Catherine W.M. Ong[1,4,5], Huilin Shao[1,2,3,8,*]

[1] Institute for Health Innovation & Technology, National University of Singapore, Singapore
[2] Department of Biomedical Engineering, Faculty of Engineering, National University of Singapore, Singapore
[3] Institute of Molecular and Cell Biology, Agency for Science, Technology and Research, Singapore
[4] Department of Medicine, Yong Loo Lin School of Medicine, National University of Singapore, Singapore
[5] Division of Infectious Diseases, Department of Medicine, National University Hospital, Singapore
[6] Department of Laboratory Medicine, National University Hospital, Singapore
[7] Department of Laboratory Medicine, Ng Teng Fong General Hospital, Singapore
[8] Department of Surgery, Yong Loo Lin School of Medicine, National University of Singapore, Singapore
[#] These authors contributed equally

* Corresponding author
Huilin Shao, PhD
National University of Singapore
MD6, 14 Medical Drive
#14-01, Singapore 117599
(65) 6601 5885
huilin.shao@nus.edu.sg

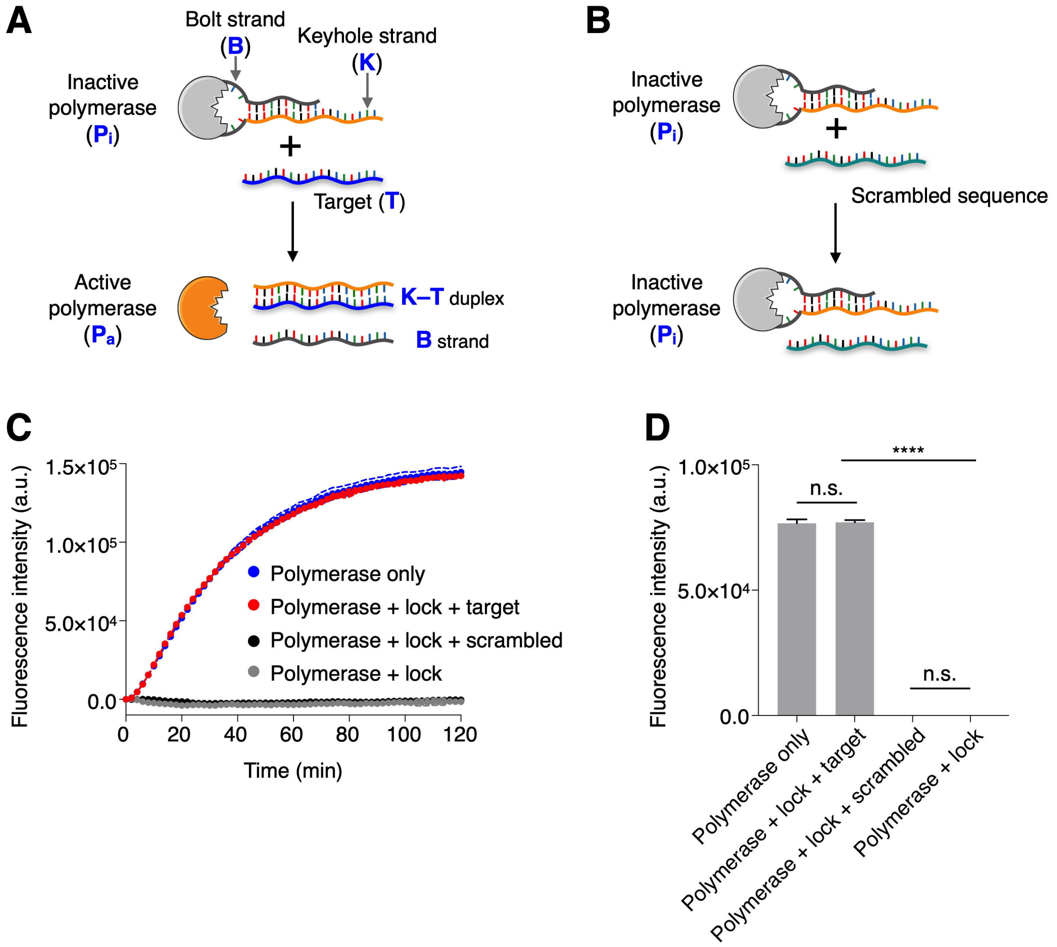

Figure S1. Polymerase activity switching by the combination lock nanostructure.
(A) The lock nanostructure binds to and inactivates DNA polymerase. Upon the addition of a specific target, the keyhole strand hybridizes with the target, releasing the bolt strand and activating the polymerase. **(B)** In the presence of a scrambled target, the keyhole strand remains assembled with the bolt strand and the polymerase, thereby keeping the polymerase inactive. **(C)** Polymerase was incubated with lock nanostructure, with or without target sequence. All resultant polymerase activity was measured in real-time by a fluorescent signaling probe. The combination lock nanostructure demonstrated potent inhibition of polymerase activity. The inhibition could be relieved through the addition of a specific target and the resultant polymerase activity recovered completely to match that of pure polymerase. **(D)** Differences in fluorescence intensity. After a 30-minute incubation, polymerase activity recovered fully in the presence of specific target. The scrambled sequence did not produce any appreciable signal. All measurements were performed in triplicate and the data are presented as mean ± s.d. a.u., arbitrary unit. (****$P < 0.0001$, n.s., not significant, Student's *t*-test).

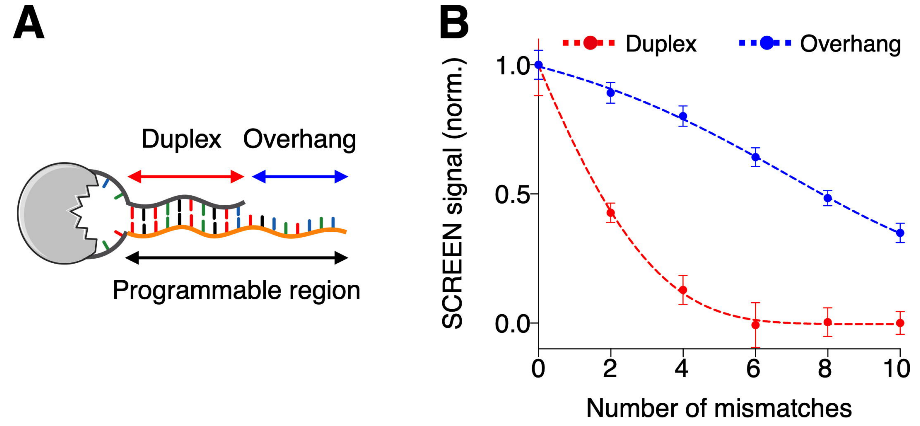

Figure S2. Specificity of the SCREEN molecular lock.

(A) Schematic of the programmable region of the lock nanostructure. The programmable region, which can be adapted to accommodate different target sequences, comprises a duplex DNA segment and a single-stranded overhang segment. **(B)** Effects of target mismatches. Synthetic nucleic acid targets, designed to have varying numbers of mismatches against the duplex and the overhang segment, respectively, were evaluated by the SCREEN platform. All measurements were performed in triplicate, and the data are presented as mean ± s.d.

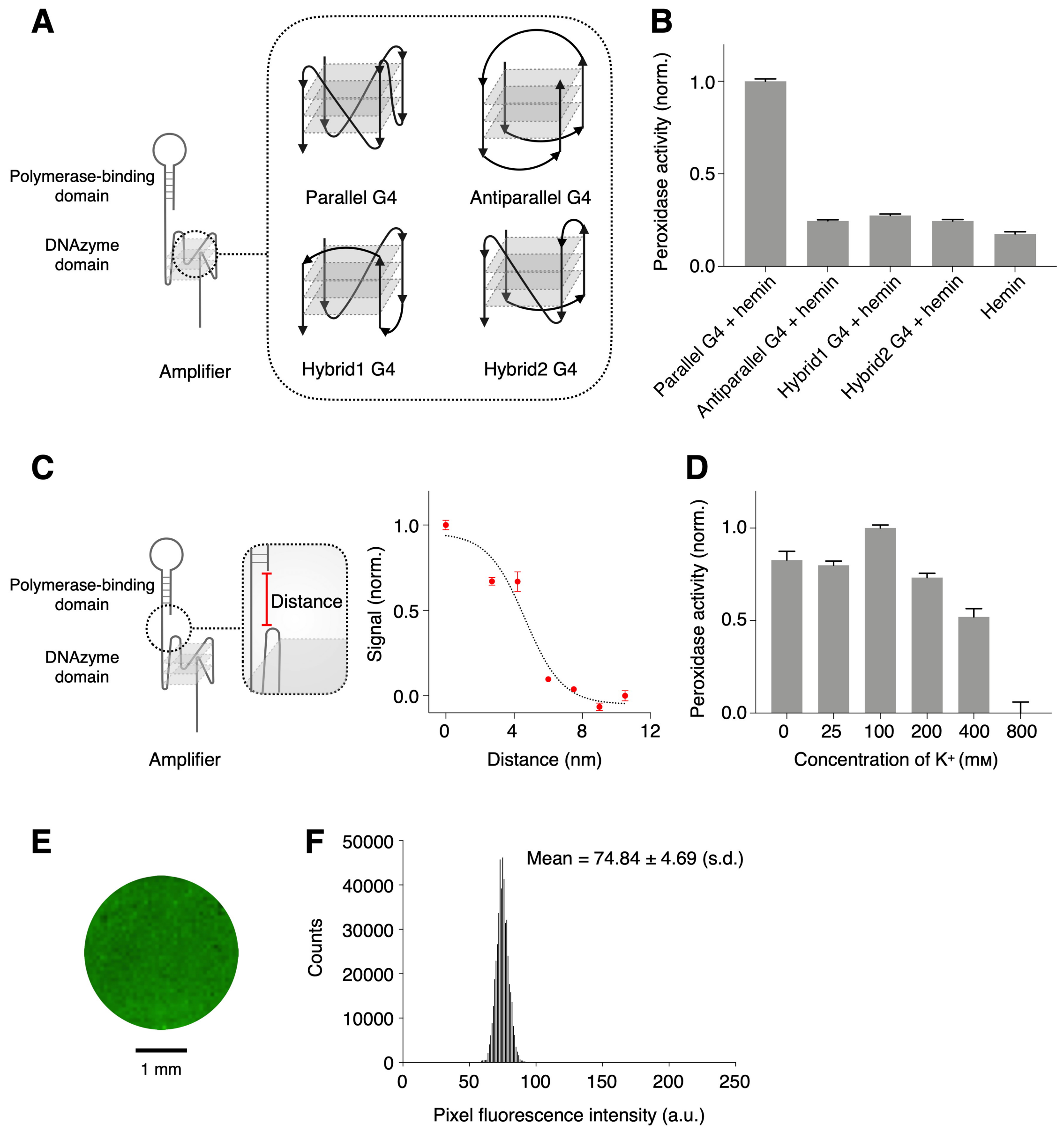

Figure S3. Design and characterization of the amplifier.
(A) Schematic representation of the amplifier strand. Various forms of G-quadruplex (G4) DNA structures (i.e., parallel, antiparallel and hybrid structures) were integrated and assessed. **(B)** Peroxidase activity of different forms of G4 structures (DNAzymes). Different G4 structures were assembled and coupled with hemin. Strong peroxidase activity was exhibited by the parallel G4 structure, which we adopted for all subsequent experiments. **(C)** Distance between the polymerase-binding domain and the DNAzyme G4 domain. Amplifier strands with a varying distance between the polymerase-binding domain and the DNAzyme domain were incubated with a fixed amount of polymerase. Resultant changes in the peroxidase activity were measured. **(D)** Amplifier stabilization by potassium ion. Peroxidase activity was measured to determine the amplifier stability. **(E)** Fluorescence image of the sensing surface functionalized with fluorophore-modified amplifier nanostructures. **(F)** Distribution of fluorescence intensity across image pixels, indicating uniform surface functionalization. All measurements were performed in triplicate and the data are presented as mean ± s.d.

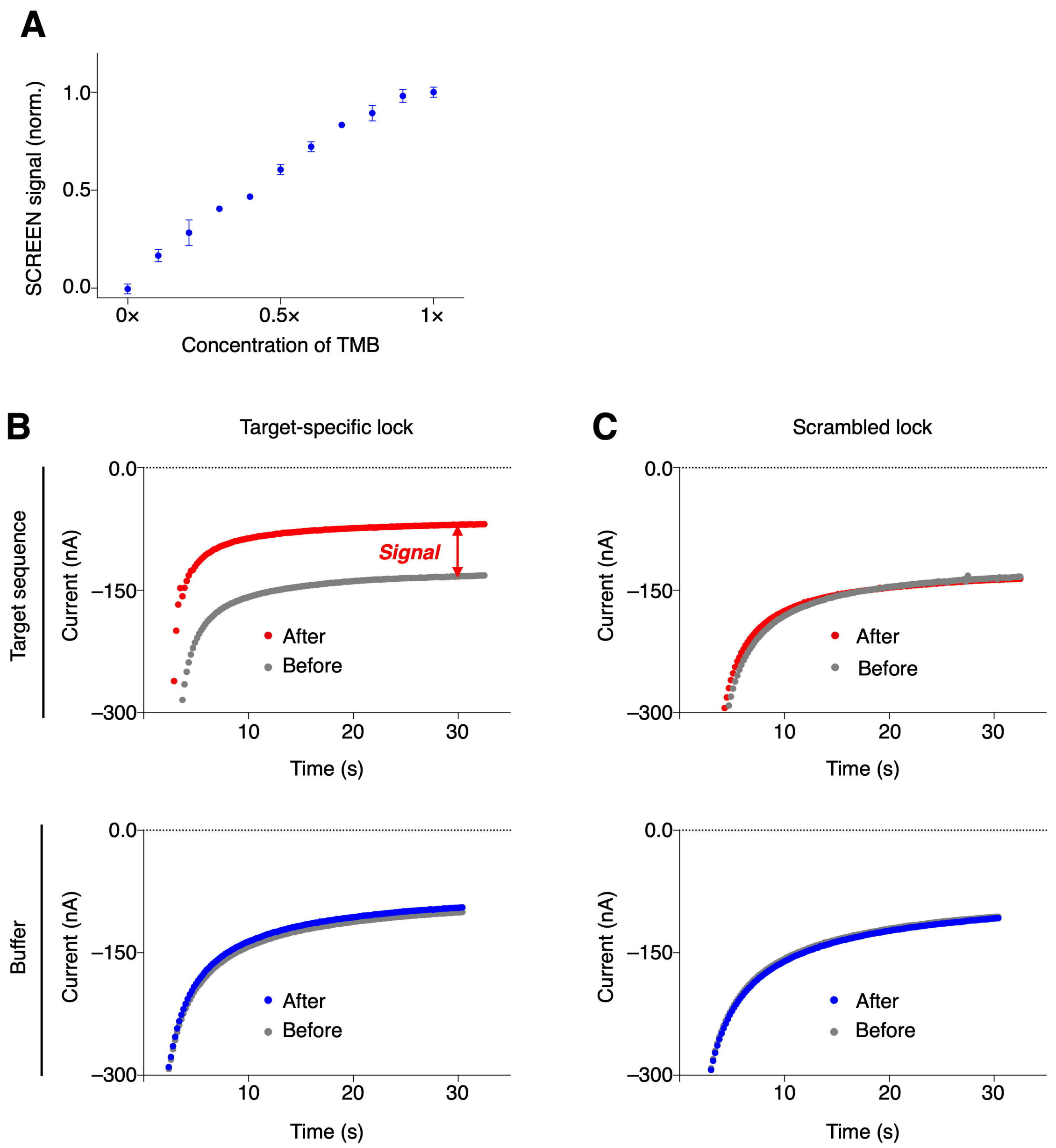

Figure S4. SCREEN electrochemical readout.

(A) Optimization of TMB concentration. Commercial TMB solution was serially diluted, used as the electrochemical substrate and the resultant signals were measured. All measurements were performed in triplicate, and the data are presented as mean ± s.d. SCREEN systems with **(B)** target-specific and **(C)** scrambled lock nanostructures were respectively incubated with samples containing target sequence or buffer only. Electrochemical readings were recorded before sample addition (baseline measurement) and after sample addition. When incubated with buffer, both lock systems showed negligible signal changes. When incubated with target sequence, the specific lock demonstrated a marked change in current, while the scrambled lock showed no appreciable signal change.

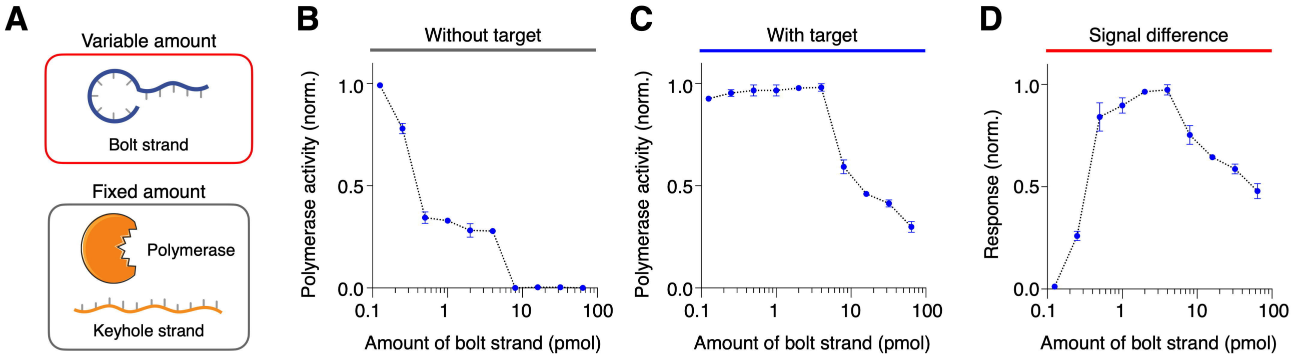

Figure S5. Characterization of complex lock mixture.
(A) Composition of complex lock mixture. To investigate the dynamics of the target recognition network, we prepared mixtures of fully-formed and partially-formed combination lock nanostructures by varying the amount of bolt strand while fixing the amounts of polymerase and keyhole strand. These mixtures were then incubated with **(B)** buffer without target or **(C)** an equal amount of target sequences. Polymerase activity was measured across all tested mixtures. **(D)** The resultant change in polymerase activity was determined as the system response, which showed differences in the extent of target-induced equilibrium shifts. All measurements were performed in triplicate and the data are presented as mean ± s.d.

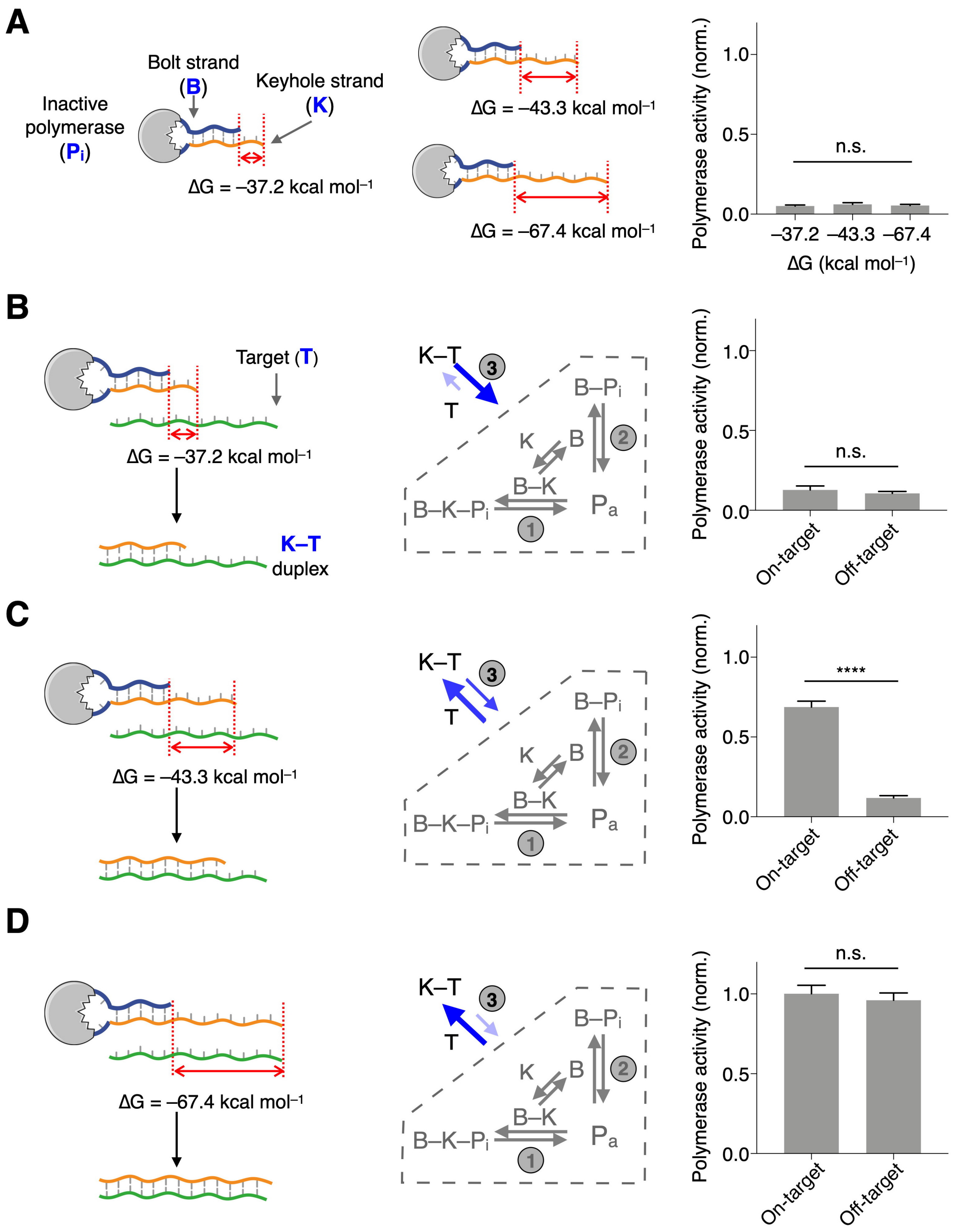

Figure S6. Response of complex lock mixture to target.

(A) Lock nanostructures with varying target affinity were prepared by changing the length of the overhang portion of the keyhole strand, thus changing the Gibbs free energy for target hybridization (ΔG). When these locks were incubated with a fixed amount of polymerase, the resultant polymerase activity showed minimal differences, indicating that the keyhole strand changes (i.e., the overhang portion) do not significantly affect the polymerase inhibition capability of the combination lock (n.s., not significant, one-way ANOVA). **(B–D)** Resultant polymerase activity when affinity-tuned lock nanostructures were incubated with on-target or off-target sequences. The blue arrows indicate the direction and extent of the equilibrium shifts. The different lock affinities affected the extent of equilibrium shifting within the complex lock mixture. All measurements were performed in triplicate and the data are presented as mean ± s.d. (****$P <$ 0.0001, n.s., not significant, Student's *t*-test).

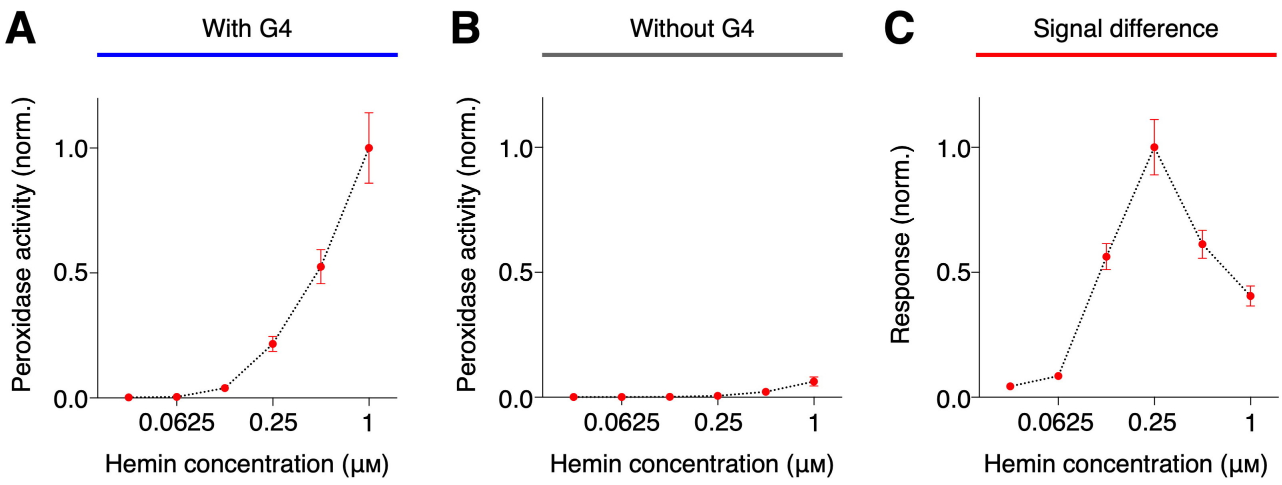

Figure S7. DNAzyme characterization.

(A) To investigate the dynamics of DNAzyme assembly, we incubated a varying concentration of hemin with a fixed concentration of G-quadruplex DNA and the resultant peroxidase activity was measured. **(B)** To provide the background signal, similar measurements were performed in the absence of G-quadruplex DNA. **(C)** While hemin alone showed weak peroxidase activity, its binding with G-quadruplex DNA gave rise to strong signal. All measurements were performed in triplicate and the data are presented as mean ± s.d.

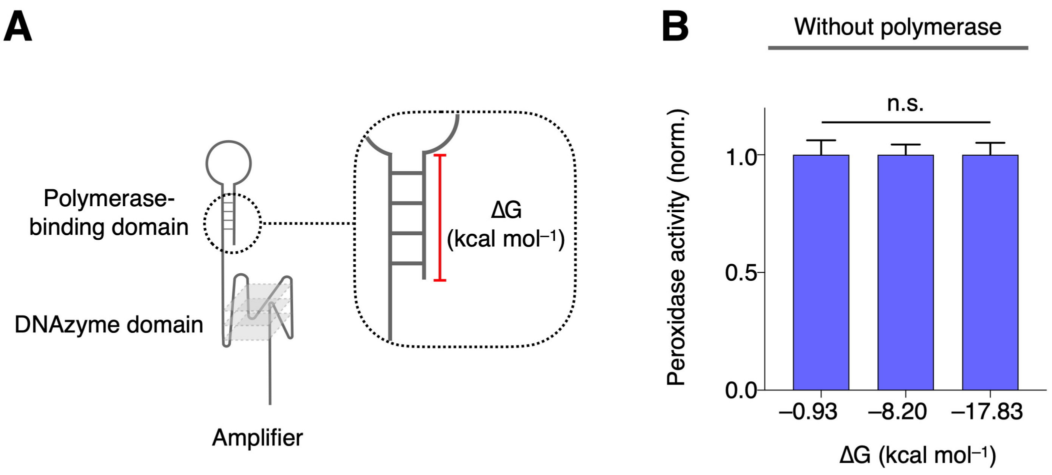

Figure S8. The polymerase-binding domain and DNAzyme activity.
(A) Scheme of the amplifier strand. The inset shows the primed section of the amplifier strand (i.e., the polymerase-binding domain), which could be varied in length (thus ΔG) to tune its priming capability. **(B)** DNAzyme peroxidase activity of different amplifier strands with varying priming capabilities. In the absence of polymerase, there was no significant difference in DNAzyme activity among all amplifier strands. All measurements were performed in triplicate and the data are presented as mean ± s.d. (n.s., not significant, one-way ANOVA).

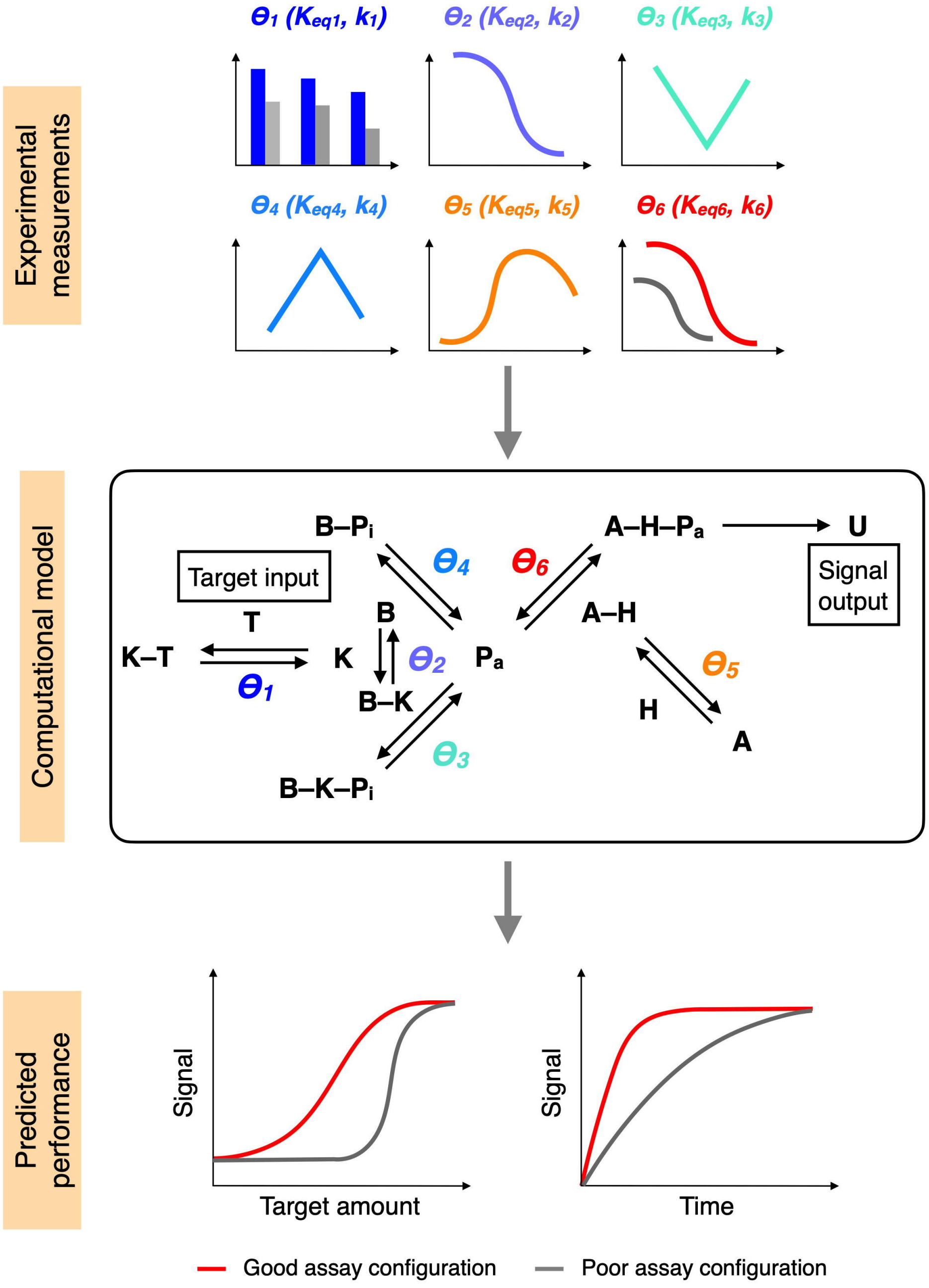

Figure S9. The integrated SCREEN model.

We determined the equilibrium and kinetic parameters (Θ_i) of individual reactions, by experimentally measuring the equilibrium (K_{eqi}) and rate (k_i) constants of respective interactions within the equilibrium network. Using these parameters to regulate the concentration changes of individual molecular components, we developed a computational model to reflect the relationships between and among reactions within the SCREEN network. The model not only enables tuning of individual molecular components, but also predicts overall assay performance of different assay configurations. By simulating various network compositions and predicting target-induced signal output, we applied the model to evaluate the overall performance of different assay configurations.

A

1. $K + T \rightleftharpoons K\text{–}T$
2. $B + K \rightleftharpoons B\text{–}K$
3. $B\text{–}K + P_a \rightleftharpoons B\text{–}K\text{–}P_i$
4. $B + P_a \rightleftharpoons B\text{–}P_i$
5. $A + H \rightleftharpoons A\text{–}H$
6. $P_a + A\text{–}H \rightleftharpoons A\text{–}H\text{–}P_a$

Equlibrium constant [M^{-1}]		Rate constant [$M^{-1}\ s^{-1}$]	
K_{eq1}	3.81E+06	k_1	6.98E+04
K_{eq2}	2.99E+06	k_2	1.17E+04
K_{eq3}	1.45E+08	k_3	8.15E+04
K_{eq4}	3.49E+05	k_4	1.37E+04
K_{eq5}	8.69E+07	k_5	7.69E+05
K_{eq6}	8.99E+06	k_6	5.26E+03

B

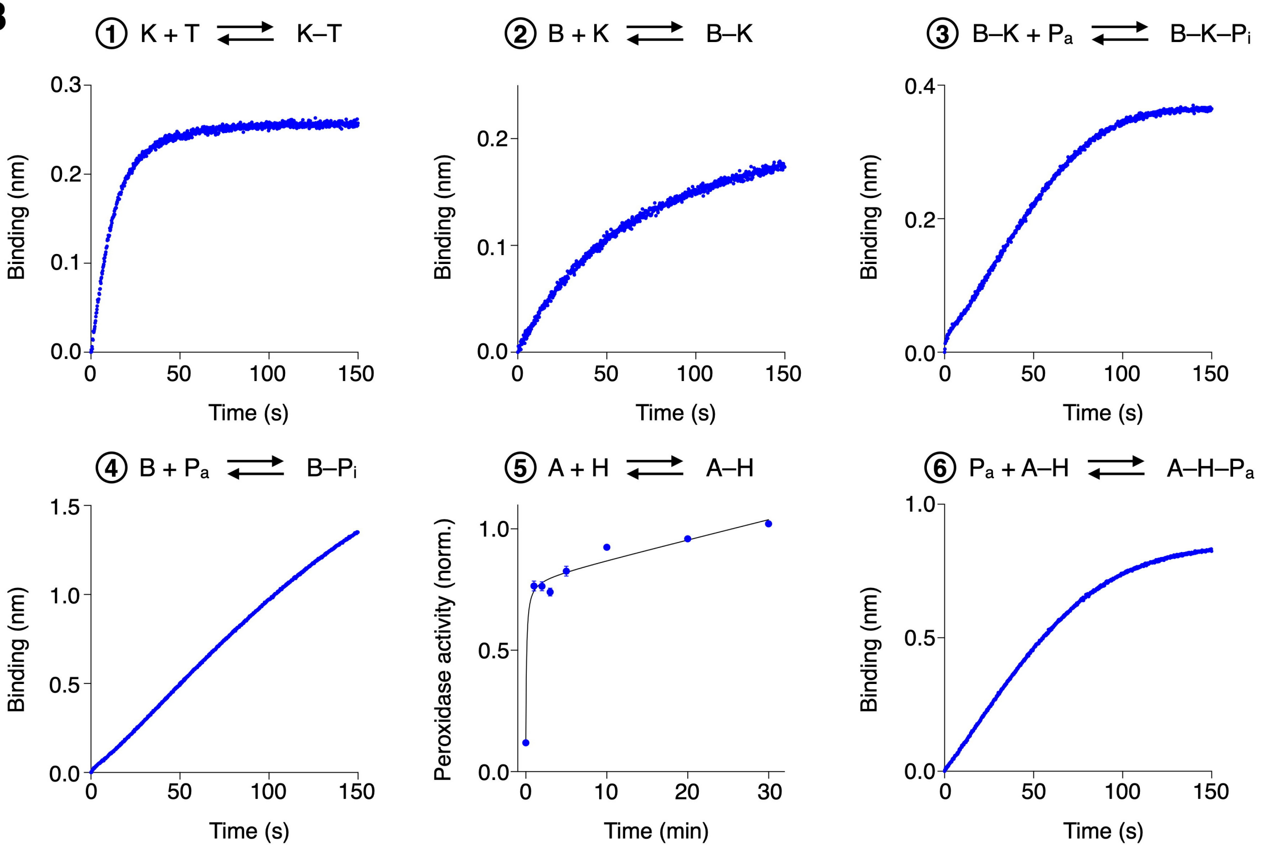

Figure S10. Equilibrium and kinetic characterization.

(A) Equilibrium and kinetic constants of reactions in the SCREEN system designed to recognize the S gene of SARS-CoV-2. For oligonucleotide hybridization (reactions 1 and 2), equilibrium constants at 25 °C were determined using the van't Hoff equation. For all other reactions, equilibrium constants were determined experimentally by calculating the reaction quotient at equilibrium. **(B)** Kinetic measurements. For reactions 1–4 and 6, real-time binding sensorgrams were obtained through biolayer interferometry. Changes in optical thickness of the biolayer were measured in a continuous manner and the curves were fitted to determine respective binding kinetics. For reaction 5, the binding of amplifier strand and hemin was monitored in a time-course experiment where the two components were mixed for different durations. Measurements were performed in triplicate and the data are presented as mean ± s.d.

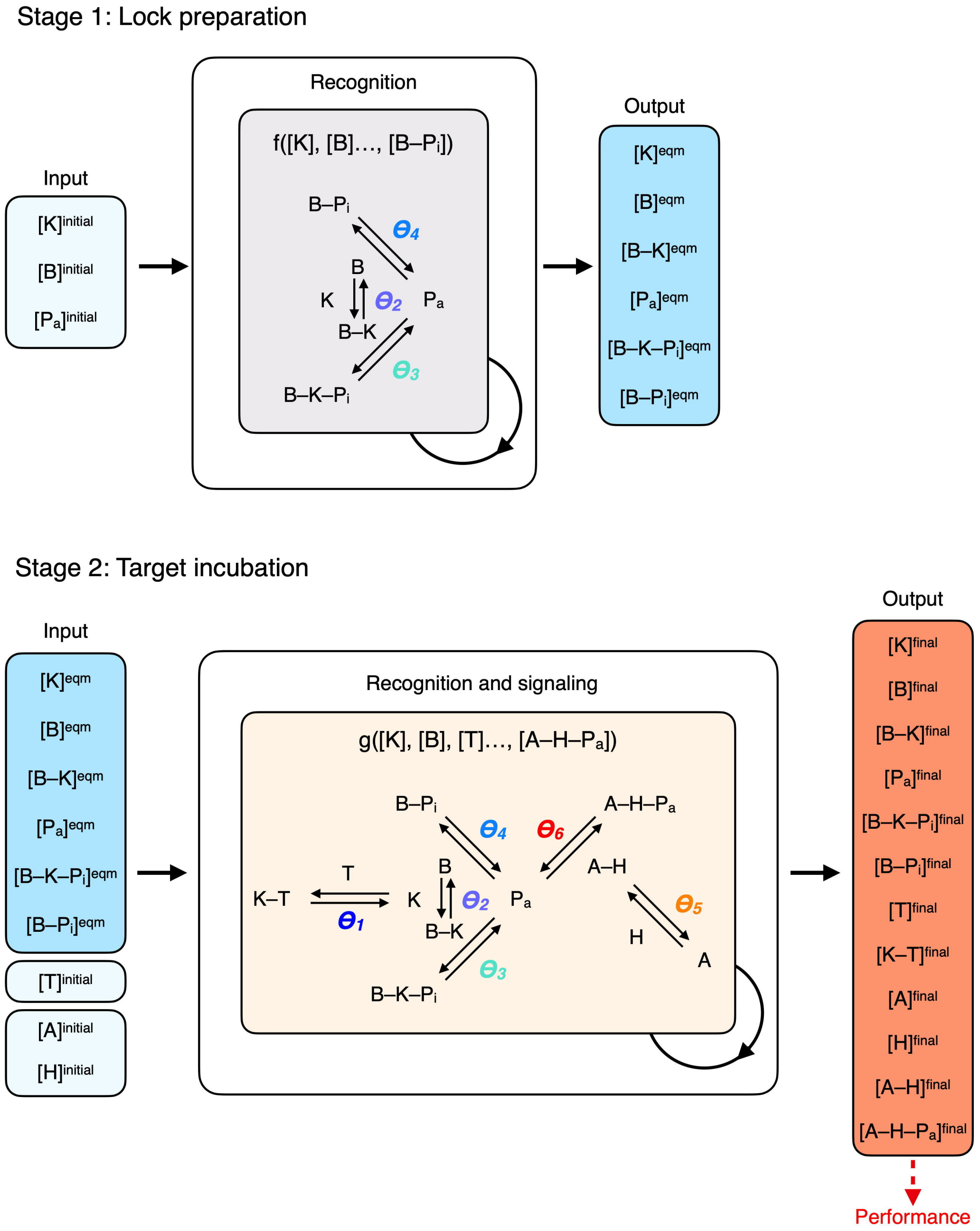

Figure S11. SCREEN simulation architecture.
The simulation was performed via a two-stage process to reflect the experimental workflow of 1) lock nanostructure preparation, and 2) target incubation with the prepared mixture for signal generation. In the first stage, in the absence of target, we used different concentrations of the molecular lock constituents (i.e., $[K]^{initial}$, $[B]^{initial}$ and $[P_a]^{initial}$) as inputs to initialize the simulation. Using the equilibrium and kinetic parameters that characterize the target recognition network (Θ_2, Θ_3 and Θ_4), we iteratively equilibrated the network. Within a single cycle, all concentrations were resolved simultaneously; for cycle propagation, the computed concentrations from the previous cycle were used as inputs for the current cycle. The process was repeated to reach a steady state (e.g., $[K]^{eqm}$, $[B]^{eqm}$). In the second stage, we used the concentration outputs from the first process (e.g., $[K]^{eqm}$, $[B]^{eqm}$) to initialize an expanded network of reactions, which comprised target, target recognition reactions and signal amplification reactions (Θ_1 – Θ_6). By varying the input concentration of target ($[T]^{initial}$), we determined the concentration of output ($[A–H–P_a]^{final}$) to evaluate the assay performance.

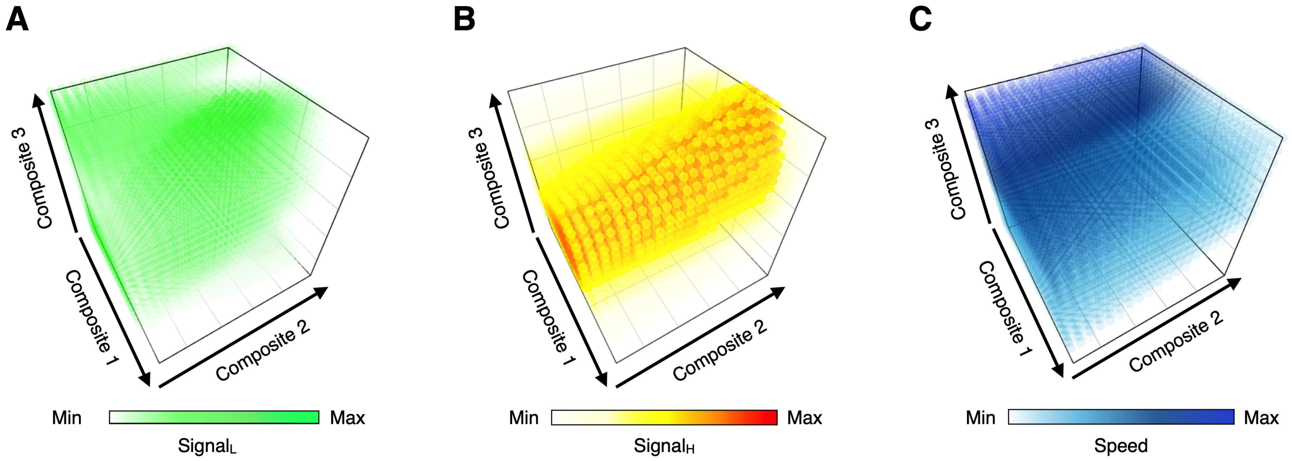

Figure S12. Simulated individual performance parameters.
(A) Signal at a low target amount ($Signal_L$), **(B)** signal at a high target amount ($Signal_H$) and **(C)** speed to reach system equilibrium by different assay configurations.

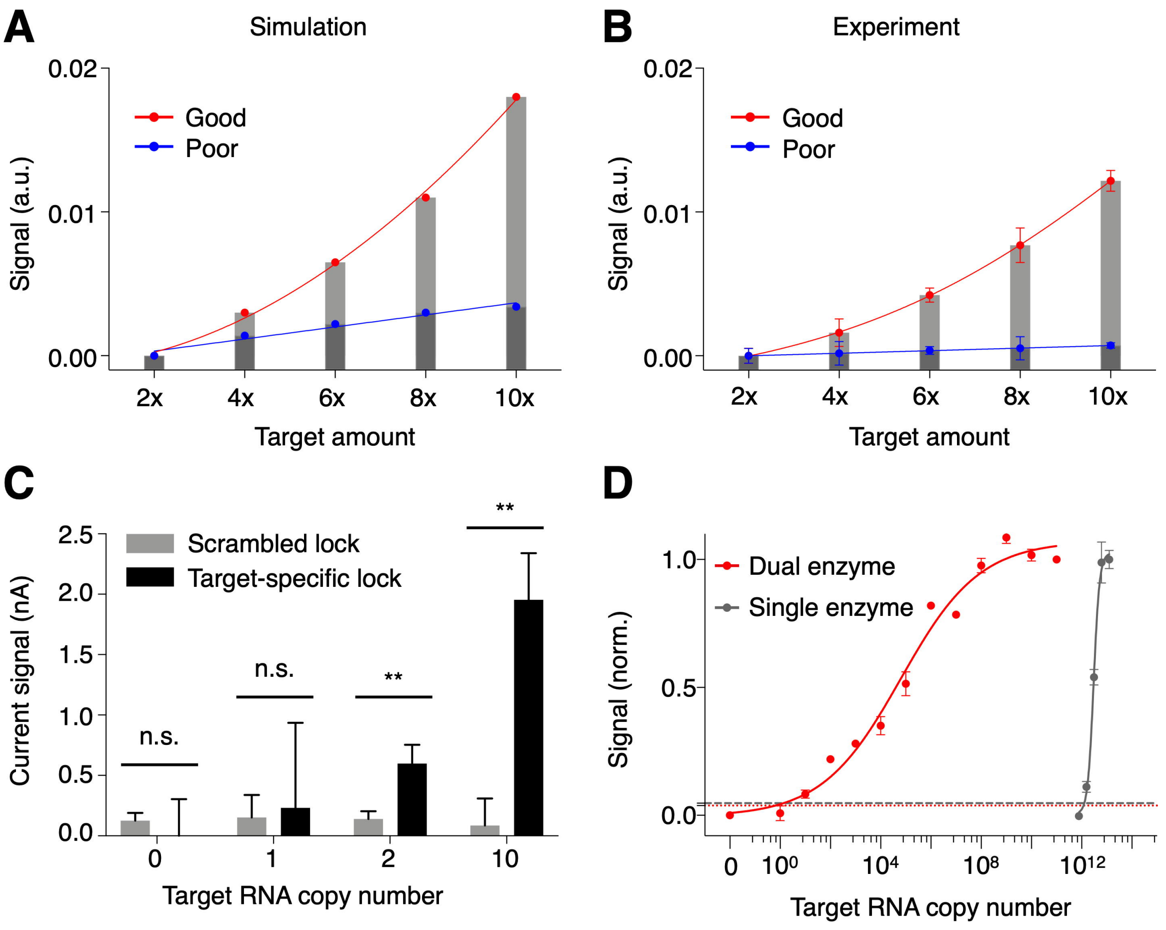

Figure S13. SCREEN signal enhancement.

(A) Simulated and **(B)** experimental measurement of target-induced response of representative good and poor performance assay configurations. Measurements were performed by introducing different amounts of target sequences (x = 10 copies). The good performance assay showed non-linear signal enhancement, especially in samples with a low copy number of target sequences. **(C)** SCREEN performance with low target amounts. Samples with low RNA copies (0, 1, 2 and 10 copies) were measured using target-specific and scrambled locks. In the single-copy measurements, we observed mean differences and large standard deviations; the standard deviation is consistent with known challenges in preparing single-copy samples (i.e., sampling errors). To reduce these sampling challenges and better characterize the detection limit, we determined the detection limit as 3 × s.d. above the SCREEN signal of the no-target sample (**Figure 3D**). This determination bypasses sampling challenges. Signals above this detection limit are considered distinguishable from the blank with >99% confidence. **(D)** Amplification efficiencies of single and dual enzyme catalysis. Target titration analyses by dual-enzyme SCREEN and single-enzyme polymerase activity. Target-induced polymerase activity (single-enzyme) was measured through 5' exonuclease degradation of fluorescent probes. The SCREEN detection limit (red dotted line) and single-enzyme detection limit (grey dotted line) were determined as 3 × s.d. above the respective signals of the no-target controls. All experimental measurements were performed in triplicate, and the data are presented as mean ± s.d. (**$P < 0.005$, n.s., not significant, Student's *t*-test).

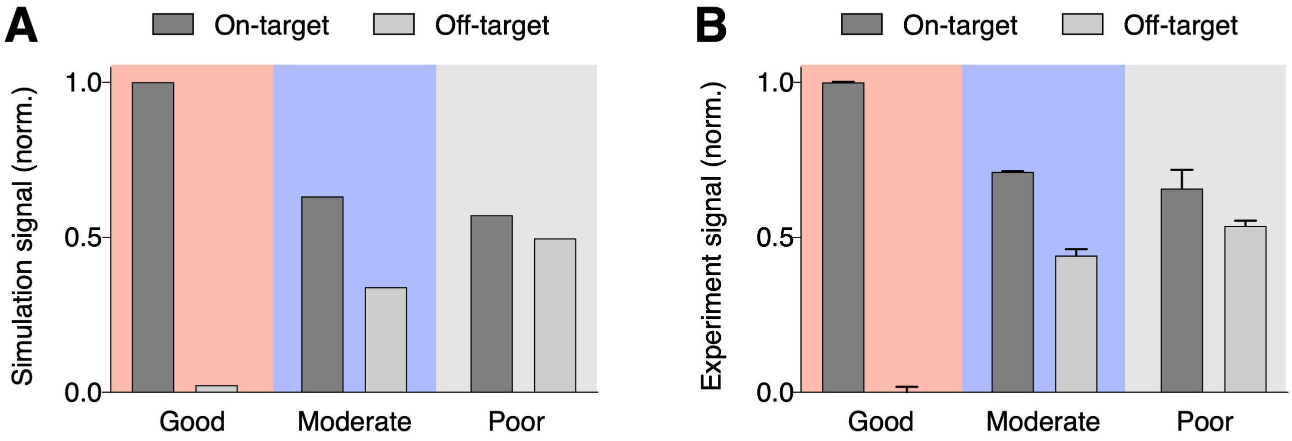

Figure S14. On- and off-target signals generated by representative assays.
(A) Simulated and **(B)** experimental signals of assays when incubated with on- and off-target sequences. Representative good, moderate and poor performance assay configurations, as determined by the computational model, were experimentally implemented. All measurements were performed in triplicate and the data are presented as mean ± s.d.

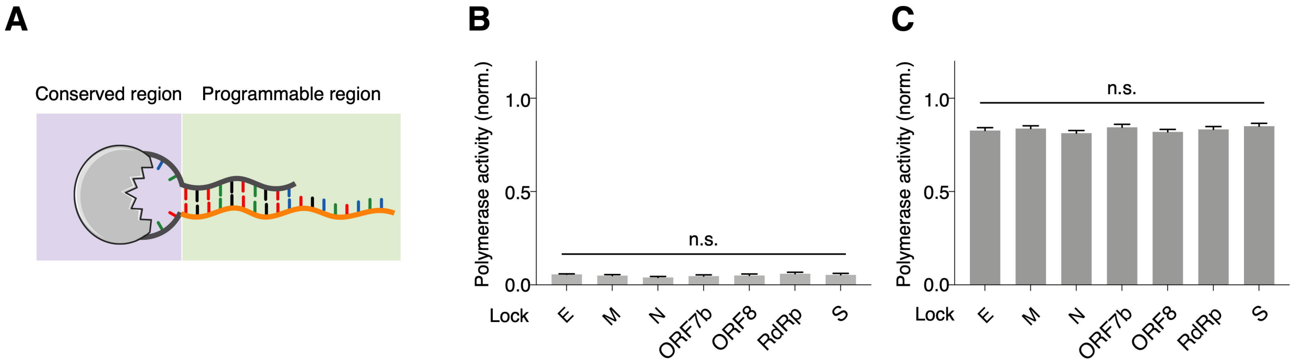

Figure S15. Programmability of the combination lock nanostructure.
(A) Schematic representation of the conserved and programmable regions of the combination lock nanostructure. The programmable region can be designed to accommodate specific target sequences of interest. **(B)** Inhibitory effect of the lock nanostructure. In the absence of target sequences, locks designed against respective SARS-CoV-2 targets (i.e., the spike (S), the envelope (E), membrane glycoprotein (M), nucleocapsid (N), open reading frame 7b (ORF7b), open reading frame 8 (ORF8) and the RNA-dependent RNA polymerase (RdRp) genes) provided strong and comparable polymerase inhibition. **(C)** Resultant polymerase activity after target incubation. Polymerase activity was well-recovered (>80%) for all locks upon the addition of their respective targets, with no significant difference in performance between locks. All measurements were performed in triplicate and the data are presented as mean ± s.d. (n.s., not significant, one-way ANOVA).

E (envelope) gene

Homology	Virus	Sequence
100%	SARS-CoV-2	TGTGCGTACTGCTGCAATATTGTTAACGTGAG----------TCTTGTAAAACCTTCTTTTTACGTTTACTCTCGTGTTAA
90.1%	SARS-CoV	TGTGCGTACTGCTGCAATATTGTTAACGTGAG----------TTTAGTAAAACCAACGGTTTACGTCTACTCGCGTGTTAA
33.3%	MERS-CoV	TGTTTCCACTGTTTTCGTGCC--TGCAACGCGCGATTCAGTTCCTCTTCACATAATCGCCCCGAGCTCGCTTATCGTTTAA
45.6%	229E	---ATTAAGCTTTGTTTCACT--TGCCATATG----------TTTTGTAATAGAA-------CAGTTTATGGCCCCATTAA
44.4%%	HKU1	TGTATTCAAATTTGTGGTTTT--TGTAATATT----------TTTATTA----TTTCACCTTCTGCCTA--TGTTTATAAT
44.4%%	NL63	TGTTTTACTTGTCATTATTTT--------------------TTTAGTAGGACA------TTATATCAACCAGTTTATAAA
46.9%	OC43	TGTATTCAACTTTGCGGTATG--TGTAATACC-------------TTAGTACTGTCCCCTTCTATTTA--TGTGTTTAAT

M (membrane glycoprotein) gene

Homology	Virus	Sequence
100%	SARS-CoV-2	GCTTCTAGA-AAGTGAACTCGTAATCGGAGCTGTGATCCTTCGTGGACATCTTCGTATTGCTGGACACCATCTAGGACGC
77.5%	SARS-CoV	GCTCATGGA-AAGTGAACTTGTCATTGGTGCTGTGATCATTCGTGGTCACTTGCGAATGGCCGGACACCCCCTAGGGCGC
48.8%	MERS-CoV	ACTCGTAGAGGACTCTACCAGTG-TAACTGCTGTTGTAACCAATGGCCACCTCAAAATGGCTGGCATGCATTTCGGTGCT
37.5%	229E	CATTCAACAAGCTCCAACAGGCATT-ACTGTGACCTTGTTGAGCGGCGTGCTTTACGTTGACGGACATAGATTGGCTTCA
45.0%	HKU1	AGTTATTGA-GGACTATCATACATTAACGGCTACTGTTATCCGTGGTCATCTTTATATACAGGGTGTTAAACTTGGCACT
43.8%	NL63	GGTGATGGCTGCACCTACAGGTATT-ACATTAACACTTCTTAGTGGTGTACTTCTTGTTGATGGCCATAAGATTGCTACT
42.5%	OC43	GATAATTGA-GGACTATCATACTCTGACGGTCACAATAATACGCGGCCATCTTTACATTCAAGGTATAAAACTAGGTACT

N (nucleocapsid phosphoprotein) gene

Homology	Virus	Sequence
100%	SARS-CoV-2	AGATTTGGATGATTTCTCCAAACAATTGCAACAATC----------CATGAGCAGTG------CTGACTCAAC-TCAGGCC
76.5%	SARS-CoV	TGACATGGATGATTTCTCCAGACAACTTCAAAATTC----------CATGAGTGGAGCTTCTGCTGATTCAAC-TCAGGCA
54.3%	MERS-CoV	TGCAAGGTAGCATCACTCAGCGCACTCGCACCCGTC----------CAAGTGTTCAGC-----CTGGTCCAATGATTGATG
28.4%	229E	TAATTAACATGATCCCTTG----------------CTTTGGCTTGACAAGGAT----------CTAGTCTTATACACAATG
39.5%	HKU1	TACTCTTGATGATCCTTATG------TAGAAGACTCTGTTGCTTAATGAGAATGAATC-----CTAATTCGACACTAGGTG
33.3%	NL63	GAATGTTTATTATTATTAGTTGCAACCCCATGCGTTTAGCGCATGATAAGGGT----------TTAGTCTTACACACAATG
46.9%	OC43	CTATACTGAAGACACCTCAG---------AAATATA----------AGAGAATGAACC-----TTATGTCGGCATCTGGTG

ORF7b gene

Homology	Virus	Sequence
100%	SARS-CoV-2	TATTCCTTGTTTTAATTATGCTTATTATCTTTTGGTTCTCACTTGAACTGCAAGATCATAATGAAACTTGTCACGCCTAA
78.5%	SARS-CoV	TATTCCTTGTTTTAATAATGCTTATTATATTTTGGTTTTCACTCGAAATCCAGGATCTAGAAGAACCTTGTA---CCAAA
10.0%	MERS-CoV	--ATCCCCTGCTGCACCT------------
6.3%	229E	---TACAGTCAAA--------------------------
35.0%	HKU1	--------------------TTATACTCCCGGTCATCATGCTGGAAGTAGAAGCTCCTC-TGGAAATCGTTCAGGAATC
10.0%	NL63	---TAATGTAAAT------------
30.0%	OC43	--------------------TTTTACTCCTGGTAAGCAATCCAGTAGTAGAGCGTCCTC-TGGAAATCGTTCTGGTAAT

ORF8 gene

Homology	Virus	Sequence
100%	SARS-CoV-2	ACAATTAATTGCCAGGAACCTAAATTGGGTAGTCTTGTAGTGCGTTGTTCGTT-------------CTATGAAGACTTTTT
29.6%	SARS-CoV	CACCTAATGTTACTATCAACTGTCAAGATCCAGCTGGTGGTGCGCTTATAGCTAGGTGTTGGTACCTTCATGAAGGTCACC
27.2%	MERS-CoV	---------------------------------------CGTGCTGTTTCCT-------------TTGCCGA--------
27.2%	229E	---------------------------------------TGGGCTGATGCAT-------------CT-------------
29.6%	HKU1	---------------------------------------TGGGTTGACCAAT-------------CTGAGCG--------
22.2%	NL63	---------------------------------------TGGGCCGATGACA----------------------------
29.6%	OC43	---------------------------------------TGGGCCGATCAGT-------------CCGACCAGTTTAG--

RdRp (RNA-dependent RNA polymerase) gene

Homology	Virus	Sequence
100%	SARS-CoV-2	ATATTAACCTTGACCAGGGCTTTAACTGCAGAGTCACATGTTGACACTGA----CTTAACAAAGCCTTACATTAAGTGGGA
70.3%	SARS-CoV	ATCCTCACTTTGACTAGGGCATTGGCTGCTGAGTCCCATATGGATGCTGA---TCTCGCAAAACCACT-TATTAAGTGGGA
58.0%	MERS-CoV	GTGCTCTCAATGACCGATTGTCTGGCCGCTGAGACACATAGGGATTGTGA---TTTTAATAAACCACT-CATTGAGTGGCC
46.9%	229E	GTTATGGGTATGACTAATTGTTTAGCTAGTGAGTGCTTTATGAAAAGTGACATCTTTGGTCAAGACTT-CAAAACTTTTGA
43.2%	HKU1	ATGTTGACTATGTGTCATGTATTAGATTGTGAATTATTTGTTAATGATAG------TTATAGACAATT----------CGA
45.7%	NL63	ATTATGGGTTTAACTAATTGTTTAGCTAGTGAGTGTTTTGTCAAGAGTGATATTTTTGGTAGTGATTT-TAAAACTTTTGA
44.4%	OC43	ATGCTGACCATGTGTCATGCATTGGATTGCGAATTGTATGTGAATAATGC------TTATAGACTATT----------TGA

S (spike) gene

Homology	Virus	Sequence
100%	SARS-CoV-2	AATAGGTATTAACATCACTAGGTTTCAAACTTTACTTGCTTTACATAGAAGTTATTTGACTCCTGGTGATTCTTCTTCAGG
14.8%	SARS-CoV	------------------------------CCTTTTCACCTGCTCAAG----------ACATTTGGGGC------------
30.9%	MERS-CoV	AATATGTTTCAATTT-------------G-CCACCTTGCCTGTTTATGATACTATT--AAGTATTATTC------------
30.9%	229E	TATCGCTATT--------------------TCTCTTTAGGTGATGTAGAAGCCGTT--AATTTCAATGT------------
37.0%	HKU1	TGGCTGTATT--------------------TTCATTT---TTATCAAGAACGTGGT--GTTTTTTATGCATATTATGCAGA
34.6%	NL63	TTTAAGTATT--------------------TTGATTTGGGTTTTATCGAAGCTGTC--AATTTTAATGT------------
43.2%	OC43	TATTTGTATT--------------------TTCATTT---TTATCAAGAAGGTGGT--ACTTTTTATGCATATTTTACAGA

Figure S16. Sequence homology of SARS-CoV-2 targets with genes of other viruses. Lock nanostructures were designed to target regions of seven different genes of SARS-CoV-2. The target sequences are tabulated. Off-target sequences derived from other viruses genes and their respective calculated homology to SARS-CoV-2 are also presented.

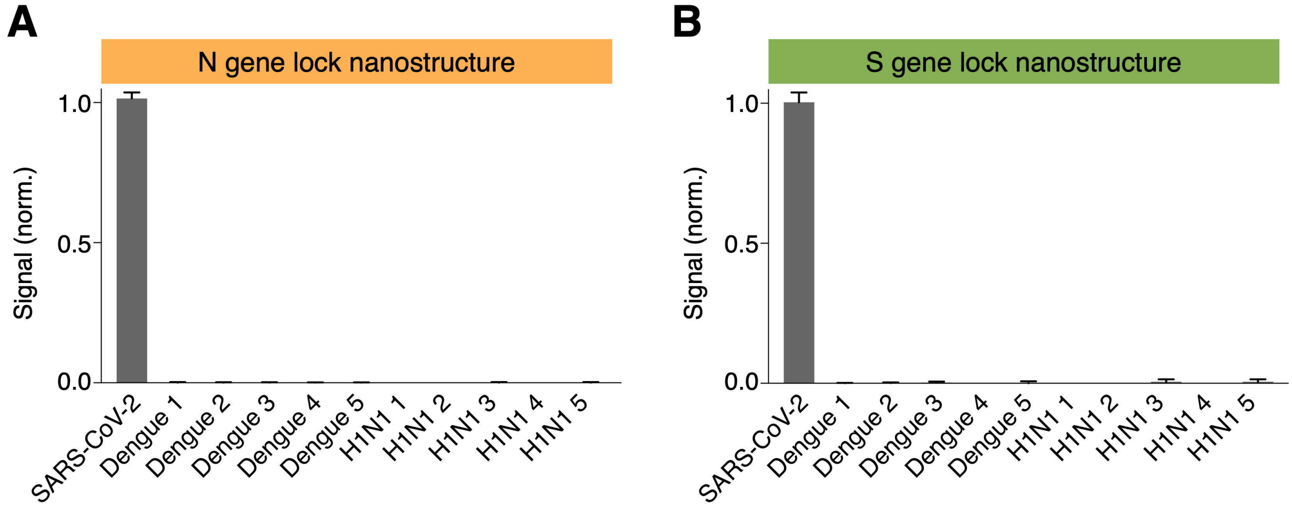

Figure S17. Specificity of SCREEN assays.

Performance of the **(A)** N gene and **(B)** S gene assays. When incubated with different nucleic acid sequences, the assays demonstrated a high signal for their respective target sequences of SARS-CoV-2, but showed negligible signals to sequences of other viruses (dengue and H1N1). Dengue and H1N1 gene sequences with the highest similarity with the SARS-CoV-2 regions of interest were selected. All measurements were performed in triplicate and the data are presented as mean ± s.d.

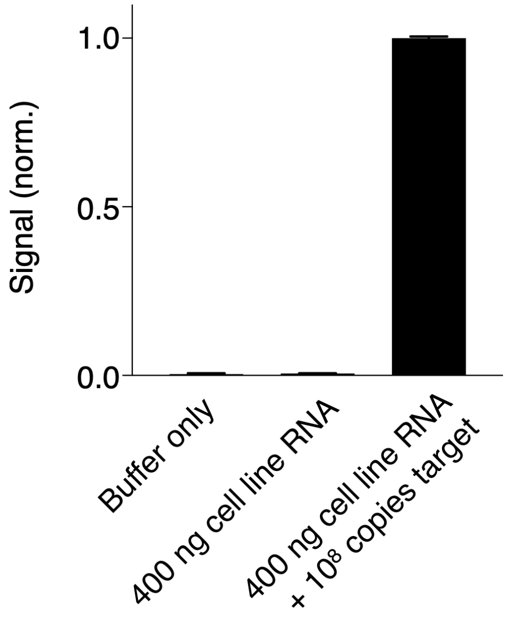

Figure S18. Robustness of the SCREEN assay against biological background.
SCREEN assay (S gene) was applied to measure buffer only, extracted cell line RNA (derived from PC9 culture) as well as synthetic viral target sequences spiked in extracted cell line RNA. The assay demonstrated specific signal against complex biological background. All measurements were performed in six replicates and the data are presented as mean ± s.d.

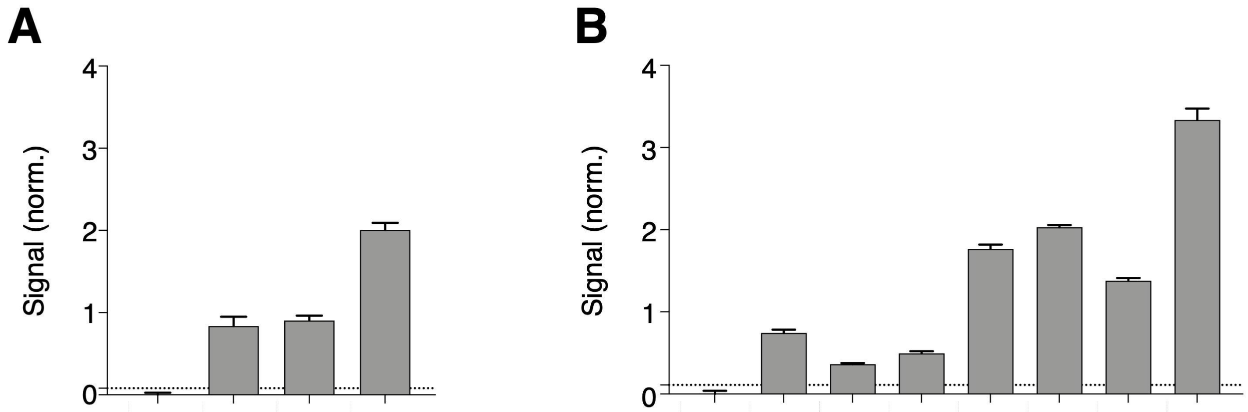

N gene	0	1	0	1
S gene	0	0	1	1

N gene	0	1	0	0	1	1	0	1
RdRp gene	0	0	1	0	1	0	1	1
S gene	0	0	0	1	0	1	1	1

Figure S19. Multiplexed target detection.
SCREEN assays were configured to measure **(A)** two or **(B)** three SARS-CoV-2 gene targets simultaneously. Both assays produced positive signal in the presence of at least one of the targets. Target inputs are tabulated, with 1 denoting presence and 0 denoting absence. The dotted line indicates the limit of detection, defined as 3 × s.d. of the signal of a no-input control. All measurements were performed in triplicate and the data are presented as mean ± s.d.

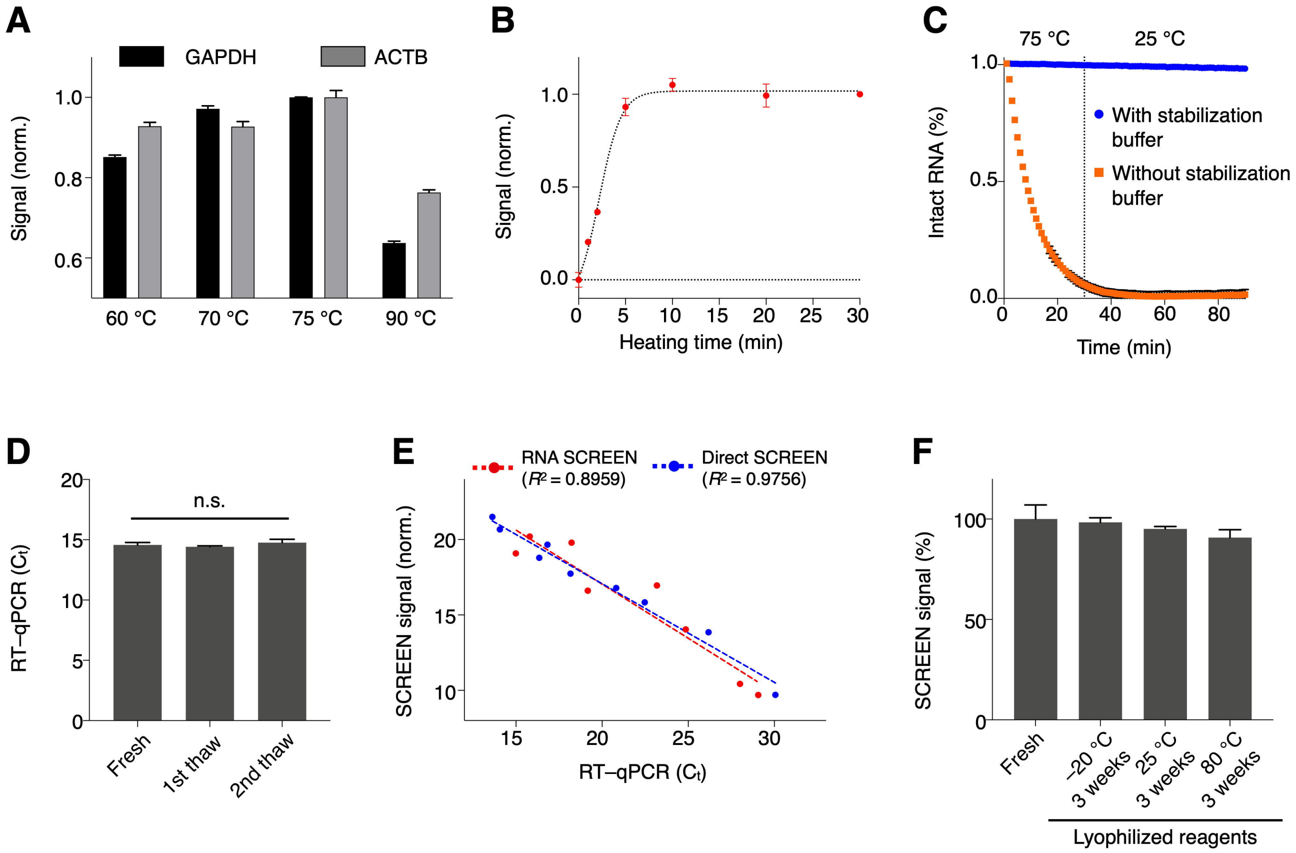

Figure S20. In vitro characterization of the SCREEN assay.

(A) Cells were heated at various temperatures for 30 minutes and the amounts of housekeeping mRNA GAPDH and beta-actin (ACTB) were measured by RT–qPCR to evaluate cellular lysis and RNA preservation. **(B)** Cells were continuously heated at 75 °C. The amount of GAPDH mRNA was measured by RT–qPCR. **(C)** Preservation of RNA integrity. An RNA probe bearing FRET pairs was spiked into cell lysate with or without the stabilization buffer. The mixture was incubated at 75 °C for 30 minutes and subsequently at 25 °C for 60 minutes. The amount of intact RNA was measured in real-time by fluorescence assay. **(D)** Effect of RNA freeze–thaw. Extracted RNA aliquots were subjected to different numbers of freeze–thaw cycles. GAPDH mRNA amounts were measured by RT–qPCR. **(E)** Correlation of SCREEN and RT–qPCR analysis. Both RNA SCREEN (R^2 = 0.8959) and direct SCREEN (R^2 = 0.9756) showed good agreement with RT–qPCR for the measurement of GAPDH and ACTB mRNA in cell line samples. **(F)** Assay stability. SCREEN assay reagents were lyophilized and stored for 3 weeks at –20 °C, 25 °C and under accelerated aging (80 °C), before being evaluated. All measurements were performed in triplicate and the data are presented as mean ± s.d.

Table S1. Oligonucleotide sequences for parameters characterization.

**Lock nanostructure**	
Spike gene bolt strand	TTATTTGACTCCTGGTGATTCAATGTACAGTATTG
Keyhole strand 1 ($\Delta G = -67.4$ kcal mol^{-1})	AATCACCAGGAGTCAAATAACTTCTATGTAAAGCAAGTAA
Keyhole strand 2 ($\Delta G = -61.5$ kcal mol^{-1})	AATCACCAGGAGTCAAATAACTTCTATGTAAAGCAA
Keyhole strand 3 ($\Delta G = -52.9$ kcal mol^{-1})	AATCACCAGGAGTCAAATAACTTCTATGTAAA
Keyhole strand 4 ($\Delta G = -43.3$ kcal mol^{-1})	AATCACCAGGAGTCAAATAACTTCTA
Keyhole strand 5 ($\Delta G = -40.7$ kcal mol^{-1})	AATCACCAGGAGTCAAATAACTTC
Keyhole strand 6 ($\Delta G = -37.2$ kcal mol^{-1})	AATCACCAGGAGTCAAATAACT
Keyhole strand 7 ($\Delta G = -34.2$ kcal mol^{-1})	AATCACCAGGAGTCAAATAA
Spike gene target	UUACUUGCUUUACAUAGAAGUUAUUUGACUCCUGGUGAUU
Spike gene off-target sequence	UAAUUCGAUGUACAUAGAAGUUAUUUGACUCCUGGUGAUU
**Amplifier**	
Amplifier 1 ($\Delta G = -17.83$ kcal mol^{-1})	CTGGGAGGGAGGGAGGGATGCTACGCATTGTCGATAGCTCTGTCGCTATC GACAATGCGT
Amplifier 2 ($\Delta G = -8.20$ kcal mol^{-1})	CTGGGAGGGAGGGAGGGATGCTACGCATTGTCGATAGCTCTGTCGCTATC GAC
Amplifier 3 ($\Delta G = -0.93$ kcal mol^{-1})	CTGGGAGGGAGGGAGGGATGCTACGCATTGTCGATAGCTCTGTC
Parallel G4	CTGGGAGGGAGGGAGGGA
Antiparallel G4	AGGGTTAGGGTTAGGGTTAGGG
Hybrid1 G4	TAGGGTTAGGGTTAGGGTTAGGG
Hybrid2 G4	AGGGTTAGGGTTAGGGTTAGGGTT

Table S2. Lock nanostructure and target sequences for SARS-CoV-2 detection.

**E (envelope) gene**	
Bolt strand	TTATTTGACTCCTGGTGATTCAATGTACAGTATTG
Keyhole strand	AGAGTAAACGTAAAAAGAAGGTTTTACAAGACTCACGTTA
SARS-CoV-2 target	GCAAUAUUGUUAACGUGAGUCUUGUAAAACCUUCUUUUUACGUUUACUCUCGUGUUAAAA
SARS-CoV sequence	GCAATAUUGUUAACGUGAGUUUAGUAAAACCAACGGUUUACGUCUACUCGCGUGUUAAAA
MERS-CoV sequence	UGCCUGCAACGCGCGAUUCAGUUCCUCUUCACAUAAUCGCCCCGAGCUCGCUUAUCGUUU
229E sequence	CUUUGUUUCACUUGCCAUAUGUUUUGUAAUAGAACAGUUUAUGGCCCCAUUAAAAAUGUG
HKU1 sequence	UUUGUGGUUUUUGUAAUAUUUUAUUAUUUCACCUUCUGCCUAUGUUUAUAAUAGAGGU
NL63 sequence	UACUUGUCAUUAUUUUUUUAGUAGGACAUUAUAUCAACCAGUUUATAAAAUUUUUCUUGC
OC43 sequence	UUUGCGGUAUGUGUAAUACCUUAGUACUGUCCCCUUCUAUUUAUGUGUUUAAUAGAGGUA
**M (membrane glycoprotein) gene**	
Bolt strand	TTCGTGGACATCTTCGTATTCAATGTACAGTATTG
Keyhole strand	AATACGAAGATGTCCACGAAGGATCACAGCTCCGATTACG
SARS-CoV-2 target	GAGGACUCUACCAGUGUAACUGCUGUUGUAACCAAUGGCCACCUCAAAAUGGCUGGCAUG
SARS-CoV sequence	GAAAGUGAACUUGUCAUUGGUGCUGUGAUCAUUCGUGGUCACUUGCGAAUGGCCGGACAC
MERS-CoV sequence	GAGGACTCTACCAGTGTAACTGCTGTTGTAACCAATGGCCACCTCAAAATGGCTGGCATG
229E sequence	AAGCUCCAACAGGCAUUACUGUGACCUUGUUGAGCGGCGUGCUUUACGUUGACGGACAUA
HKU1 sequence	AGGACUAUCAUACAUUAACGGCUACUGUUAUCCGUGGUCAUCUUUAUAUACAGGGUGUUA
NL63 sequence	GGCUGCACCUACAGGUAUUACAUUAACACUUCUUAGUGGUGUACUUCUUGUUGAUGGCCA
OC43 sequence	AGGACUAUCAUACUCUGACGGUCACAAUAAUACGCGGCCAUCUUUACAUUCAAGGUAUAA
**ORF7b gene**	
Bolt strand	GTCACGCCTAAACGAACATGCAATGTACAGTATTG
Keyhole strand	CATGTTCGTTTAGGCGTGACAAGTTTCATTATGATCTTGC
SARS-CoV-2 target	CATGTTCGTTTAGGCGTGACAAGTTTCATTATGATCTTGC
SARS-CoV sequence	UUUCACUCGAAAUCCAGGAUCUAGAAGAACCUUGUACCAAAGUCUAAACGAACAUGAAAC
MERS-CoV sequence	CGAAUCUCAAUUUCAUUGUUAUGGCAUCCCCUGCUGCACCUCGUGCUGUUUCCUUUGCCG
229E sequence	UUUUUCUAAACUGAACGAAAAGAUGGCUACAGUCAAAUGGGCUGAUGCAUCUGAACCACA
HKU1 sequence	UCAUCAUGCUGGAAGUAGAAGCUCCUCUGGAAAUCGUUCAGGAAUCCUCAAGAAAACUUC
NL63 sequence	UUUAAUCUAAACUAAACAAAAUGGCUAAUGUAAAUUGGGCCGAUGACAGAGCUGCUAGGA
OC43 sequence	GGUAAGCAAUCCAGUAGUAGAGCGUCCUCUGGAAAUCGUUCUGGUAAUGGCAUCCUCAAG
**ORF8 gene**	
Bolt strand	TAGTCTTGTAGTGCGTTGTTCAATGTACAGTATTG
Keyhole strand	AACAACGCACTACAAGACTA CCCAATTTAGGTTCCTGGCA
SARS-CoV-2 target	ACAAUUAAUUGCCAGGAACCUAAAUUGGGUAGUCUUGUAGUGCGUUGUUCGUUCUAUGAA

SARS-CoV sequence	CACCUAAUGUUACUAUCAACUGUCAAGAUCCAGCUGGUGGUGCGCUUAUAGCUAGGUGUU
MERS-CoV sequence	AUCCCCUGCUGCACCUCGUGCUGUUUCCUUUGCCGAUAACAAUGAUAUAACAAAUACAAA
229E sequence	UACAGUCAAAUGGGCUGAUGCAUCUGAACCACAACGUGGUCGUCAGGGUAGAAUACCUUA
HKU1 sequence	AAAUCGUUCAGGAAUCCUCAAGAAAACUUCUUGGGUUGACCAAUCUGAGCGAAGCCAUCA
NL63 sequence	ACUAAACAAAAUGGCUAAUGUAAAUUGGGCCGAUGACAGAGCUGCUAGGAAGAAAUUUCC
OC43 sequence	AGCGUCCUCUGGAAAUCGUUCUGGUAAUGGCAUCCUCAAGUGGGCCGAUCAGUCCGACCA
**N (nucleocapsid phosphoprotein) gene**	
Bolt strand	AATCCATGAGCAGTGCTGACCAATGTACAGTATTG
Keyhole strand	GTCAGCACTGCTCATGGATTGTTGCAATTGTTTGGAGAAA
SARS-CoV-2 target	GAUGAUUUCUCCAAACAAUUGCAACAAUCCAUGAGCAGUGCUGACUCAACUCAGGCCUAA
SARS-CoV sequence	UUCUCCAGACAACUUCAAAAUUCCAUGAGUGGAGCUUCUGCUGAUUCAACUCAGGCAUAA
MERS-CoV sequence	CAAGGUAGCAUCACUCAGCGCACUCGCACCCGUCCAAGUGUUCAGCCUGGUCCAAUGAUU
229E sequence	UUAACAUGAUCCCUUGCUUUGGCUUGACAAGGAUCUAGUCUUAUACACAAUGGUAAGCCU
HKU1 sequence	CUCUUGAUGAUCCUUAUGUAGAAGACUCUGUUGCUUAAUGAGAAUGAAUCCUAAUUCGAC
NL63 sequence	AGUUGAAUGUUUAUUAUUAUUAGUUGCAACCCCAUGCGUUUAGCGCAUGAUAAGGGUUUA
OC43 sequence	AUACUGAAGACACCUCAGAAAUAUAAGAGAAUGAACCUUAUGUCGGCAUCUGGUGGUAAC
**RdRp (RNA-dependent RNA polymerase) gene**	
Bolt strand	CATGTTGACACTGACTTAACCAATGTACAGTATTG
Keyhole strand	GTTAAGTCAGTGTCAACATGTGACTCTGCAGTTAAAGCCC
SARS-CoV-2 target	ACCUUGACCAGGGCUUUAACUGCAGAGUCACAUGUUGACACUGACUUAACAAAGCCUUAC
SARS-CoV sequence	ACUUUGACUAGGGCAUUGGCUGCUGAGUCCCAUAUGGAUGCUGAUCUCGCAAAACCACUU
MERS-CoV sequence	UCAAUGACCGAUUGUCUGGCCGCUGAGACACAUAGGGAUUGUGAUUUUAAUAAACCACUC
229E sequence	GUAUGACUAAUUGUUUAGCUAGUGAGUGCUUUAUGAAAAGUGACAUCUUUGGUCAAGACU
HKU1 sequence	GACUAUGUGUCAUGUAUUAGAUUGUGAAUUAUUUGUUAAUGAUAGUUAUAGACAAUUCGA
NL63 sequence	GGGUUUAACUAAUUGUUUAGCUAGUGAGUGUUUUGUCAAGAGUGAUAUUUUUGGUAGUGA
OC43 sequence	GACCAUGUGUCAUGCAUUGGAUUGCGAAUUGUAUGUGAAUAAUGCUUAUAGACUAUUUGA
**S (spike) gene**	
Bolt strand	TTATTTGACTCCTGGTGATTCAATGTACAGTATTG
Keyhole strand	AATCACCAGGAGTCAAATAACTTCTATGTAAAGCAAGTAA
SARS-CoV-2 target	GUUUCAAACUUUACUUGCUUUACAUAGAAGUUAUUUGACUCCUGGUGAUUCUUCUUCAGG
SARS-CoV sequence	UGGUAUUAACAUUACAAAUUUUAGAGCCAUUCUUACAGCCUUUUCACCUGCUCAAGACAU
MERS-CoV sequence	UUCAAUUUGCCACCUUGCCUGUUUAUGAUACUAUUAAGUAUUAUUCUAUCAUUCCUCACA
229E sequence	AUCGCUAUUUCUCUUUAGGUGAUGUAGAAGCCGUUAAUUUCAAUGUCACUAAUGCUGCAA

HKU1 sequence	GGCUGUAUUUCAUUUUUAUCAAGAACGUGGUGUUUUUAUGCAUAUAUGCAGAUGUAG
NL63 sequence	UUAAGUAUUUUGAUUUGGGUUUUAUCGAAGCUGUCAAUUUUAAUGUCACGACAGCUAGUG
OC43 sequence	UUAAGUAUUUUGAUUUGGGUUUUAUCGAAGCUGUCAAUUUUAAUGUCACGACAGCUAGUG
**GAPDH gene**	
Bolt strand	GACAACAGCCTCAAGATCATCAATGTACAGTATTG
Keyhole strand	ATGATCTTGAGGCTGTTGTCATACTTCTCATGGTTCACAC
Target	GUGUGAACCAUGAGAAGUAUGACAACAGCCUCAAGAUCAU
**Beta-actin (ACTB) gene**	
Bolt strand	TGCAAGGCCGGCTTCGCGGGCAATGTACAGTATTG
Keyhole strand	CCCGCGAAGCCGGCCTTGCACATGCCGGAGCCGTTGTCGA
Target	UCGACAACGGCUCCGGCAUGUGCAAGGCCGGCUUCGCGGG
**H1N1 sequences**	
H1N1 1	UUGUUGAACGCAGCAAAGCCUACAGCAACUGUUACCCUUA
H1N1 2	CAUAUGGGGCCUGUCCCAGAUAUGUUAAGCAAAACACUCU
H1N1 3	UACAGGCAAUCUCCAAACAUUGAAGAUAAGAGUACAUGAG
H1N1 4	AAUAGGCAAGUCAUAGUUGACAGAGGUAAUAGGUCCGGUU
H1N1 5	UACCAGAUUUGUAUGAUUACAAGGAGAAUAGAUUCAUCGA
**Dengue sequences**	
Dengue 1	AAAUGCCCCCGGAUCACUGAGACGGAACCAGAUGACGUUG
Dengue 2	CCCAGAAAGGGAUCAUUUUUAUUUUGCUGAUGCUGGUAAC
Dengue 3	UUGAAGACGGAGGUCACAAACCCUGCCGUCCUGCGCAAAC
Dengue 4	UUGCACUAUGCAUGGAAGACAAUGGCUAUGAUACUGUCAA
Dengue 5	AAAAAUUCUAAAUCCCUAUAUGCCGAGUGUGGUAGAAACU

Table S3. Comparison of SARS-CoV-2 nucleic acid detection technologies.

	SCREEN	CRISPR[1,2]	LAMP[3,4]	Sequencing[5,6]	RT–qPCR[7,8]
**RNA extraction**	No (direct SCREEN)	Some	Some	Yes	Yes
**Reverse transcription**	No	Some	Yes	Yes	Yes
**Target amplification**	No	Yes	Yes	Yes	Yes
**Sequence design stringency**	Low	Moderate	High	Moderate–high	Moderate–high
**Detection limit [copies per reaction]**	1	42–100	10–100	10–50	<10
**Assay duration (samples-to-results)**	As little as 35 min	30 min–3 h	1.5–2.5 h	6–24 h	2–4 h
**Reaction temperature**	Isothermal (room temperature)	Isothermal	Isothermal	Thermal cycling	Thermal cycling
**Equipment requirement**	Low	Low	Low	High	Moderate

References:

[1] J. P. Broughton, X. Deng, G. Yu, C. L. Fasching, V. Servellita, J. Singh, X. Miao, J. A. Streithorst, A. Granados, A. Sotomayor-Gonzalez, K. Zorn, A. Gopez, E. Hsu, W. Gu, S. Miller, C. Y. Pan, H. Guevara, D. A. Wadford, J. S. Chen & C. Y. Chiu, *Nat Biotechnol.* **2020**, *38*, 870.

[2] M. Patchsung, K. Jantarug, A. Pattama, K. Aphicho, S. Suraritdechachai, P. Meesawat, K. Sappakhaw, N. Leelahakorn, T. Ruenkam, T. Wongsatit, N. Athipanyasilp, B. Eiamthong, B. Lakkanasirorat, T. Phoodokmai, N. Niljianskul, D. Pakotiprapha, S. Chanarat, A. Homchan, R. Tinikul, P. Kamutira, K. Phiwkaow, S. Soithongcharoen, C. Kantiwiriyawanitch, V. Pongsupasa, D. Trisrivirat, J. Jaroensuk, T. Wongnate, S.

Maenpuen, P. Chaiyen, S. Kamnerdnakta, J. Swangsri, S. Chuthapisith, Y. Sirivatanauksorn, C. Chaimayo, R. Sutthent, W. Kantakamalakul, J. Joung, A. Ladha, X. Jin, J. S. Gootenberg, O. O. Abudayyeh, F. Zhang, N. Horthongkham & C. Uttamapinant, *Nat Biomed Eng.* **2020**, *4*, 1140.
[3] G. S. Park, K. Ku, S. H. Baek, S. J. Kim, S. I. Kim, B. T. Kim & J. S. Maeng, *J Mol Diagn.* **2020**, *22*, 729.
[4] L. Yu, S. Wu, X. Hao, X. Dong, L. Mao, V. Pelechano, W. H. Chen & X. Yin, *Clin Chem.* **2020**, *66*, 975.
[5] Q. Wu, C. Suo, T. Brown, T. Wang, S. A. Teichmann & A. R. Bassett, *Sci Adv.* **2021**, *7*,
[6] M. Wang, A. Fu, B. Hu, Y. Tong, R. Liu, Z. Liu, J. Gu, B. Xiang, J. Liu, W. Jiang, G. Shen, W. Zhao, D. Men, Z. Deng, L. Yu, W. Wei, Y. Li & T. Liu, *Small.* **2020**, *16*, e2002169.
[7] B. Fung, A. Gopez, V. Servellita, S. Arevalo, C. Ho, A. Deucher, E. Thornborrow, C. Chiu & S. Miller, *J Clin Microbiol.* **2020**, *58*, e01535-20.
[8] L. Bordi, A. Piralla, E. Lalle, F. Giardina, F. Colavita, M. Tallarita, G. Sberna, F. Novazzi, S. Meschi, C. Castilletti, A. Brisci, G. Minnucci, V. Tettamanzi, F. Baldanti & M. R. Capobianchi, *J Clin Virol.* **2020**, *128*, 104416.
